# Anomalous enhancement of resurgent Na^+^ currents at high temperatures by SCN9A mutations underlies the episodic heat-enhanced pain in inherited erythromelalgia

**DOI:** 10.1038/s41598-019-48672-6

**Published:** 2019-08-22

**Authors:** Chiung-Wei Huang, Hsing-Jung Lai, Po-Yuan Huang, Ming-Jen Lee, Chung-Chin Kuo

**Affiliations:** 1 Department of Physiology, National Taiwan University College of Medicine, Taipei, Taiwan; 2 Department of Neurology, National Taiwan University Hospital Jinshan Branch, New Taipei City, Taiwan; 3 Department of Neurology, National Taiwan University Hospital, Taipei, Taiwan; 4 Department of Medical Genetics, National Taiwan University Hospital, Taipei, Taiwan; 5 Department of Physiology, Kaohsiung Medical University, Kaohsiung, Taiwan

**Keywords:** Ion channels in the nervous system, Neurophysiology

## Abstract

Inherited erythromelalgia (IEM), caused by mutations in Na_v_1.7 channel is characterized by episodic neuropathic pain triggered especially by warm temperature. However, the mechanism underlying the temperature–dependent episodic attacks of IEM remains elusive. We investigated the electrophysiological effect of temperature changes on Na_v_1.7 channels with three different mutations, p.I136V, p. I848T, and p.V1316A, both *in vitro* and *in vivo*. *In vitro b*iophysical studies of the mutant channels show consistent temperature-dependent enhancement of the relative resurgent currents if normalized to the transient currents, as well as temperature-dependent changes in the time to peak and the kinetics of decay of the resurgent currents, but no congruent temperature–dependent changes in steady–state parameters such as shift of activation/inactivation curves and changes of the absolute size of the window or resurgent currents. *In vivo* nerve excitability tests (NET) in IEM patients reveal the essentially normal indices of NET at a single stimulus. However, there are evident abnormalities if assessed with preconditioning pulses, such as the decrease of threshold elevation in hyperpolarizing threshold electrotonus (50–100 ms), the increase of inward rectification in current–voltage curve, and the increase of refractoriness at the interpulse interval of 2–6 ms in recovery cycle, probably also implicating derangements in temperature dependence of inactivation and of recovery from inactivation in the mutant channels. The pathogenesis of heat–enhanced pain in IEM could be attributed to deranged temperature dependence of Na_v_1.7 channels responsible for the genesis of resurgent currents.

## Introduction

Primary erythromelalgia or inherited erythromelalgia (IEM; OMIN 133020) is an autosomal dominant chronic neurological disorder caused by mutations in human *SCN9A* gene encoding α subunit of Na_v_1.7 channel, which is abundantly expressed in trigeminal, sympathetic, and dorsal root ganglion neurons^1–4^. IEM is clinically characterized by episodic excruciating burning pain and erythema in the limbs. The symptomatic aggravation is frequently evoked by warm stimuli and relieved by cooling^5–7^. The nature of episodic attack is not fully understood but could be responsible for the core symptomatic pathogenesis of IEM and might shed light on potential therapies for the excruciating clinical conditions.

The IEM–causing mutations are scattered throughout the whole Na_v_1.7 channel protein, resulting in different gating alterations including hyperpolarizing shift in the activation curve, slowed macroscopic deactivation kinetics, and an increase in sustained currents^8–10^. However, despite that pain is constantly exacerbated by local heat among different mutations, there are only very limited and incongruous data on the effect of temperature on channel gating. A previous study by Han *et al*. showed a similar hyperpolarizing shift of the steady–state activation curve with p.L858F mutation at either warmer or cooler temperatures^11^. From a small Taiwanese cohort of the IEM patients, three mutations of SCN9A gene, p.I136V, p.I848T, and p.V1316A have been identified^7^. The familial segregation for the p.I136V mutation was confirmed in a Taiwanese family^12^. Both p.I848T and p.V1316A mutations are from sporadic cases; but they recurred in IEM patients with different ethnic origin^7,13–15^. The frequencies from these mutant alleles are less than 0.01% and they are likely pathogenic. The p.I136V and p.V1316A mutations caused further hyperpolarizing shift in the activation curve at warmer temperature, whereas the p.I848T mutant channel showed a depolarizing shift. Thus, the core pathophysiologic mechanism for heat–induced pain in IEM could not be fully explained by changes in steady–state parameters.

Resurgent sodium currents were previously proposed to be unaltered in IEM-related mutations^16^. However, we have recently discovered a significantly increase and altered kinetics of resurgent current with p.V1316A mutation^14^. Also, the effect of temperature on gating kinetics of the Na_v_1.7 channel has not been fully characterized and may well contribute to the intriguing heat–enhanced pain exacerbation. In this study, we investigated the effect of temperature changes on the molecular behavior of the wild–type (WT) and different mutant Na_v_1.7 channels both *in vitro* and *in vivo* (with nerve excitability test, NET), endeavoring to identify the key pathophysiological elements responsible for the symptomatic pathogenesis of IEM. The *in vitro* biophysical studies demonstrate that the activation kinetics of resurgent currents are much slower at 15 °C than at 25 °C, and even more so if compared to that at 40 °C in the mutant Na_v_1.7 channels, but the temperature dependence is far less pronounced in the WT channels. Consistently, the IEM patients show increased refractoriness and reduced superexcitability in recovery cycle (RC), and reduced threshold elevation at late hyperpolarizing threshold electrotonus (TE), both implying abnormalities in the recovery from inactivation in Na^+^ channels. Moreover, these NET abnormalities are effectively ameliorated by local cooling. These findings indicate that deranged resurgent Na^+^ currents, which are consequences of deranged temperature dependence of the kinetics of relevant molecular transitions, play the major and crucial role in the pathogenesis of IEM.

## Results

### Larger window currents in the mutant Na_v_1.7 channels

**Figure 1:**
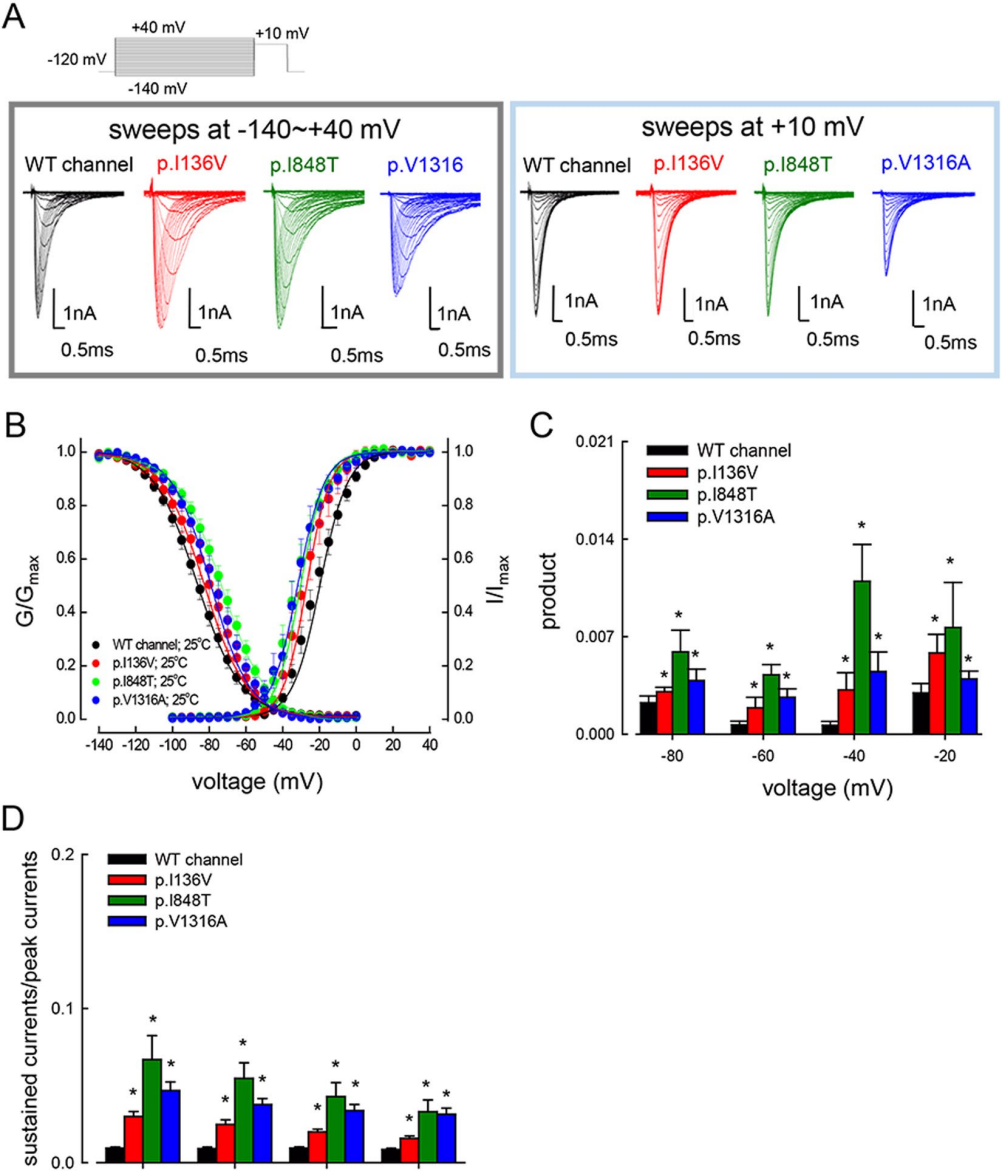
Activation and inactivation curves of WT, p.I136V, p.I848T, and p.V1316A mutant channels. (**A**) Sample sweeps for the making of the activation (left panel) and inactivation (right panel) curves of the WT, p.I136V, p.I848T, and p.V1316A mutant channels in the presence of the Na_v_β4 peptide. (**B**) The activation and inactivation curves of the WT and mutant channels at 25 °C are fitted with a Boltzmann function of the form: 1/[1 + exp((V_h_ − V)/*k*)], where V is the membrane potential, V_h_ and *k* are −21.8 ± 1.5 mV and 8.3 ± 0.43 for the activation curve, and −85.7 ± 2.17 mV and −12.5 ± 0.33 for the inactivation curve in the WT channel; −27.5 ± 1.4 mV and 7.7 ± 0.23 for the activation curve, and −80.5 ± 1.2 mV and −12.8 ± 0.21 for the inactivation curve in p.I136V mutant channel; −32.6 ± 1.3 mV and 7.6 ± 0.23 for the activation curve, and −73.5 ± 1.3 mV and −12.5 ± 0.37 for the inactivation curve in the p.I848T mutant channel; and −34.04 ± 1.47 mV and 8.7 ± 0.34 for the activation curve, and −77.1 ± 0.9 mV and −11.9 ± 0.12 for the inactivation curve in the p.V1316A mutant channel, respectively (n = 5 for each measurement). (**C**) The product of G/G_max_ and I/I_max_ at each voltage from part **B** is plotted against the voltage (i.e., −20, −40, −60, and −80 mV) in the WT, p.I136V, p.I848T, and p.V1316A mutant channels (*p < 0.05 between the WT and each mutant channel). (**D**) The ratio between the sustained (the average currents between 90 and 95 ms of the pulse) and peak (maximal) transient currents at +10 to +30 mV (see part A for the sample sweeps) is significantly larger in the p.I136V, p.I848T, and p.V1316A mutant channels in comparison with the WT channels (*p < 0.05 between the WT and each mutant channel).

The activation and inactivation curves of the p.I136V, p.I848T, and p.V1316A mutant channels are negatively and positively shifted in the membrane potential axis, respectively (Fig. 1A,B). On the other hand, the slope of both gating curves is grossly unchanged or only minimally or negligibly changed by these mutations (Fig. 1B). Figure 1C shows a closer view of the product of the two curves at each voltage (i.e., −20, −40, −60, and −80 mV), presumably a measurement of the “window current” or the sustained currents at each voltage. The products at each voltage are always larger in each of the mutant than in the wild type (WT) channels. The increase of sustained currents in the mutant channels at more positive voltages could also be directly demonstrated through measurement of the late currents during a depolarizing pulse (Fig. 1D), and could potentially contribute to the neural hyperexcitability in IEM^14^.

### Incongruent window current changes with increasing temperature from 15 °C to 40 °C

**Figure 2:**
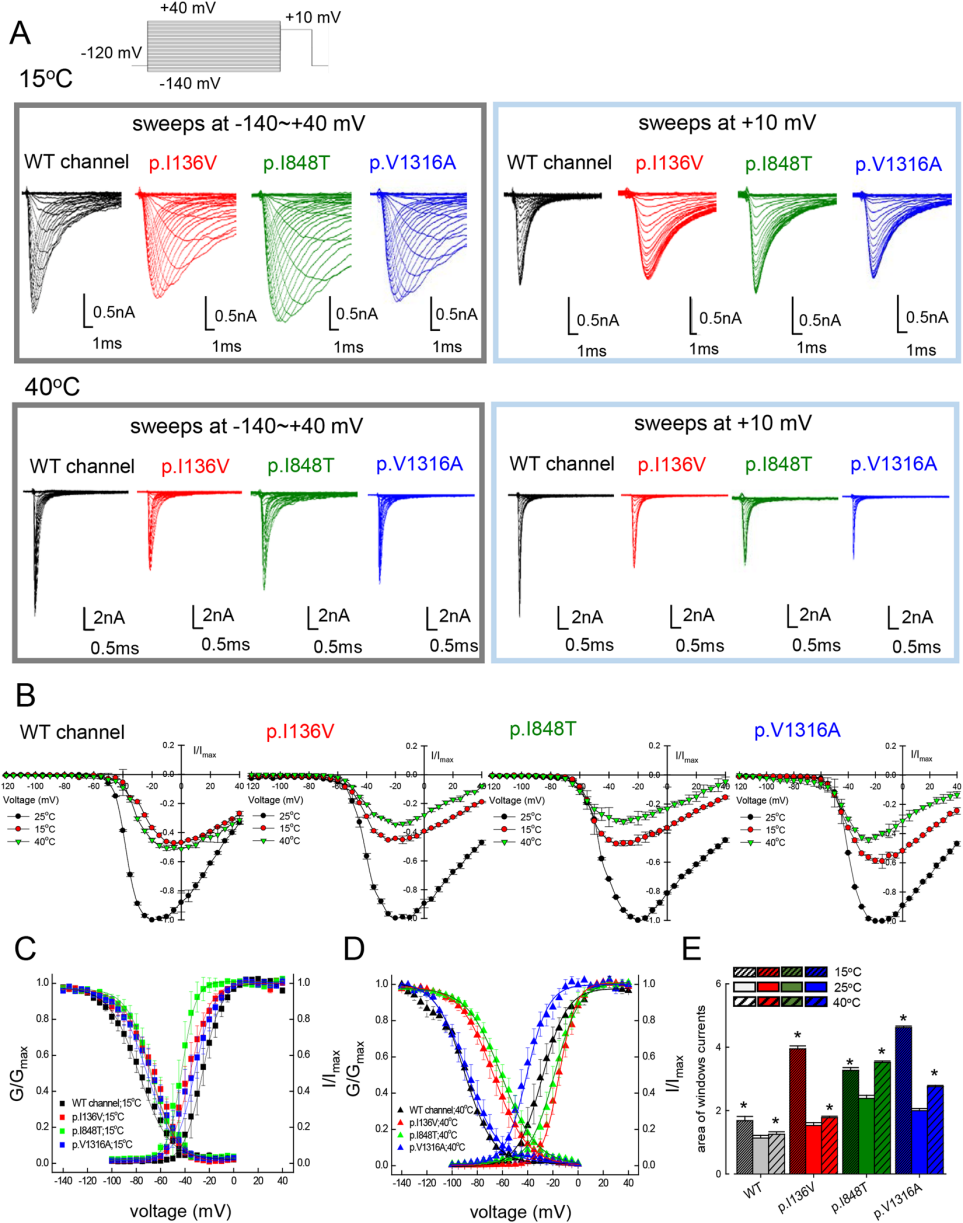
Activation and inactivation curves of WT, p.I136V, p.I848T, and p.V1316A mutant channels at 15 °C and 40 °C. (**A**) Sample sweeps for the making of the activation (left panel) and inactivation (right panel) curves of the WT, p.I136V, p.I848T, and p.V1316A mutant channels at 15 °C or 40 °C. (**B**) The normalized current-voltage (I/V) curves of the WT, p.I136V, p.I848T, and p.V1316A mutant channels at 25 °C, 15 °C, or 40 °C are constructed by plotting the normalized current (normalization to the peak currents at −20mV and 25 °C in the same cell) against membrane voltage (n = 3 for each group). (**C**) The activation and inactivation curve of the WT, p.I136V, p.I848T, and p.V1316A mutant channels at 15 °C are fitted with a Boltzmann function (n = 5 for each measurement). The sample sweeps are shown in part **A**. (**D**) The activation and inactivation curves of the WT and mutant channels at 40 °C are fitted with a Boltzmann function (n = 5 for each measurement). The sample sweeps are shown in part (**A)**. (**E**) The area of window currents is obtained from part **B** and **C** for the WT and different mutant channels at 15 °C, 25 °C, and 40 °C, respectively (*p < 0.05 compared to 25 °C in the WT and mutant channels, respectively).

Since warmth usually induces the excruciating pain in IEM patients, we assessed the effects of temperature changes on the WT, p.I136V, p.I848T, and p.V1316A mutant channels (Fig. 2A,B). Note that the peak transient currents appear to be the largest at 25 °C, and are smaller at both 15 °C and 40 °C. Lowering the medium temperature to 15 °C causes a hyperpolarizing shift of the activation curve but a depolarizing shift of the inactivation curve in the p.I136V, p.I848T, and p.V1316A mutant channels if compared with the WT channel (Fig. 2C). On the other hand, an increase of ambient temperature to 40 °C causes a depolarizing shift of the activation curve in the p.I136V, and p.I848T mutant channels, but a hyperpolarizing shift in the p.V1316A mutant channel in comparison with the WT channel (Fig. 2D). Moreover, the inactivation curves of the p.I136V and p.I848T, but not the p.V1316A mutant channels are shifted in the depolarizing direction if compared with the WT channel (Fig. 2D). All the mutant channels therefore tend to produce even larger window currents than the WT channel, both in higher (40 °C) and lower (15 °C) temperatures if compared with 25 °C (Fig. 2E and Supplementary Data Fig. S1). Increased window or sustained currents, albeit a common feature of all the mutant channels, thus does not seem to play the cardinal role in the pathogenesis of IEM, which is characterized by marked symptomatic aggravation by warming but ameliorated by cooling. In this regard, it is interesting to note that the kinetics of macroscopic inactivation (the decay of the transient currents) are much slower in the mutant than in the WT channels at 15 °C but not at 40 °C (Supplementary Data Fig. S2), signaling a higher temperature dependence or “Q_10_” in the inactivation–related kinetics with IEM–causing mutations (see below).

### Larger resurgent currents in the mutant Na_v_1.7 channel at 40 °C but not at 15 °C

**Figure 3:**
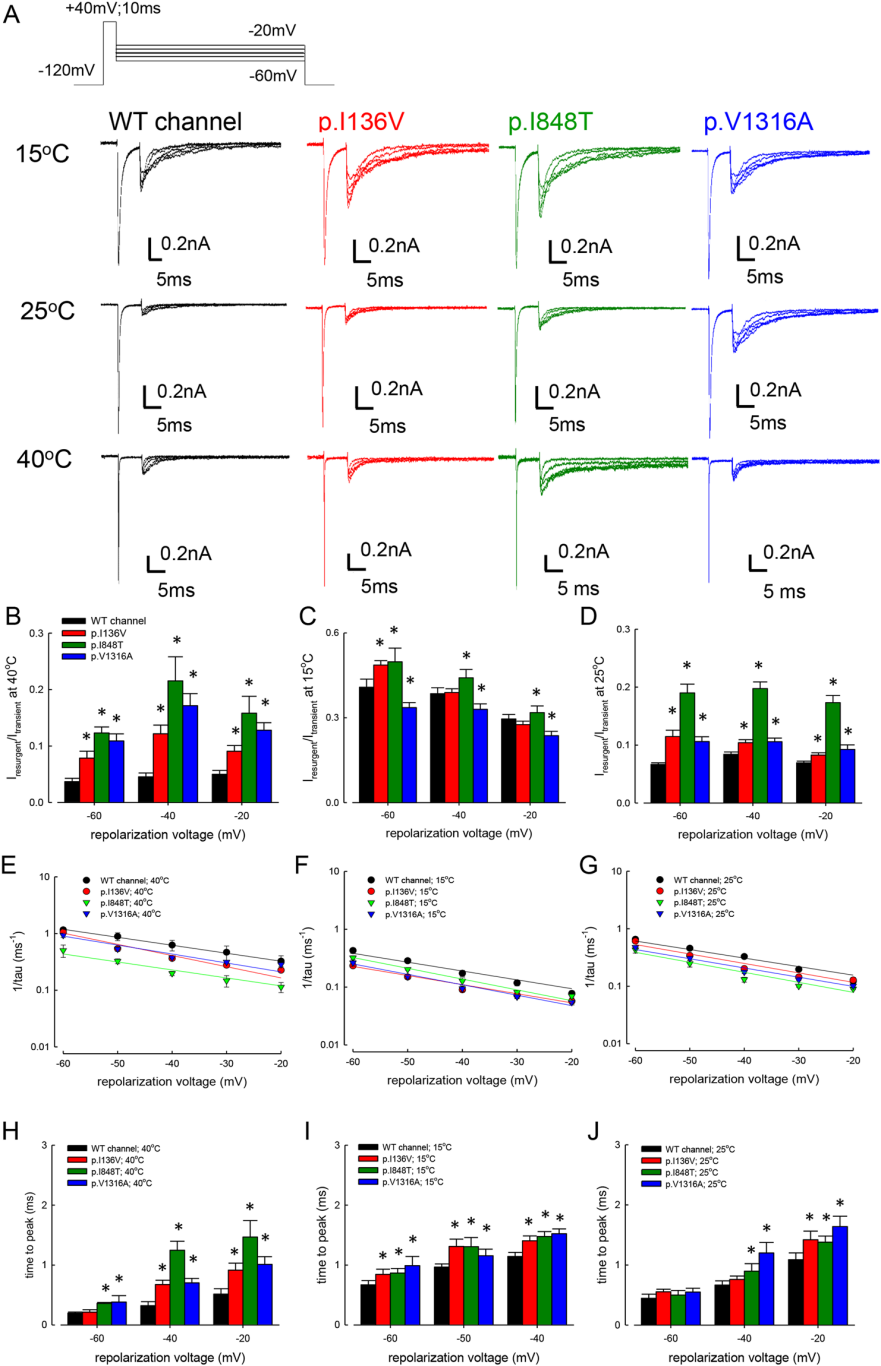
Resurgent currents of WT, p.I136V, p.I848T, and p.V1316A mutant channels at 15 °C, 25 °C and 40 °C. (**A**) The cells were held at −120 mV, and the resurgent currents were evoked by pulses between −20 mV and −60 mV in 10 mV increment after a prepulse of +40 mV for 10 ms at these different temperatures (15 °C, 25 °C, and 40 °C). Note that we have deliberately chosen a strongly depolarizing and short prepulse for a smaller decay and thus a more complete examination of the resurgent currents^14^. Also note the much faster kinetics of the currents at 40 °C than at 15 °C. (**B**) Cumulative results were obtained from the experiments described in part **A** for the WT and three mutant channels (n = 5 for each measurement). The ratio between the peak amplitude of the resurgent and transient Na^+^ currents (I_resurgent_/I_transient_) in the same cell is significantly larger in the mutant than WT channels at 40 °C. *P < 0.05. (**C**) Cumulative results were obtained from the experiments described in part **A**. The ratio between the resurgent and transient Na^+^ currents is significantly larger in the p.I136V, p.I848T, but smaller in the p.V1316A mutant than WT channels at 15 °C (n = 5 for each measurement). *P < 0.05. (**D**) Cumulative results were obtained from the experiments described in part (**A**) (n = 5 for each measurement). The ratio between resurgent and transient Na^+^ currents is significant larger in the mutant than in the WT channels at 25 °C. *P < 0.05. **(E**–**G**) Cumulative results were obtained from the same experiments described in part **A** (n = 5 for each measurement). The inverses of decay time constants (1/tau (ms^−1^)) of the resurgent currents plotted against the voltage (mV) in semi–logarithmic scales. The lines are linear regressions of the form: 1/tau_(V)_ = A × exp (*k* × V/25) ms^−1^. The voltage dependence of the decay kinetics is rather similar in the WT and mutant channels at the three different temperatures. (**H**–**J**) Cumulative results were obtained from the experiments described in part **A** (n = 5 for each measurement). Note that the time to peak of resurgent currents (the time from the end of depolarization prepulse of +40 mV to the resurgent current peak at different potentials) in the mutant channels is evidently longer than that in the WT channels with the repolarization potentials between −20 and −60 mV at 40 °C (right panel), but the difference gets smaller at 25 °C (middle panel) and especially at 15 °C (left panel). *p < 0.05 by Student’s independent *t* test.

We have shown that the resurgent currents are significantly larger at room temperature in the p.V1316A mutant channel than in the WT channel^14^. Consistently, the resurgent currents appear to be larger in all the three mutants than in the WT channel at 25 °C (Fig. 3A). On the othe hand, the absolute size of the resurgent currents seems to be the largest in 15 °C, apparently inconsistent with the relief of pain and reduced nerve excitability in IEM in low temperatures. However, a closer examination reveals that the normalized resurgent currents (I_resurgent_) and the area under the resurgent current (Q_resurgent_) (normalized to the transient currents in the same cell) in all the mutant channels are most enhanced at 40 °C but least enhanced at 15 °C if compared with that in the WT channel (Fig. 3B–D and Supplementary Data Fig. S3). Because size of the resurgent currents is determined by the rates of entry to and exit from the resurgent open state^14^, we investigated changes in the kinetics of the resurgent currents in different temperatures. The decay gets faster at higher temperatures in all channels. However, the decay is always slower in the mutant channels, and such a difference is more prominent at higher temperatures (Fig. 3E–G). Also, the differences in time to peak between WT and the mutant channels are much larger at 40 °C than at 15 °C (Fig. 3H–J). We also examined the activation curve of resurgent currents and the kinetics of genesis of the inactivated states during the depolarization prepulse (which is indirectly related to the making of resurgent currents during the subsequent repolarization) in different temperatures for the WT and mutant channels. There is no congruent or consistent trend of changes in the activation curves of the WT, p.I136V, p.I848T, and p.V1316A mutant channels at 15 °C, 25 °C, and 40 °C (Supplementary Data Fig. S4). The resurgent current became smaller with lengthening of the depolarization prepulse in the WT and all mutant channels (Supplementary Data Fig. S5). The rate of decay was always slightly (~2–fold) slower in the three mutant channels than in the WT channel at both 15 °C and 25 °C at the same prepulse voltage. The steady–state relative residual resurgent currents, on the other hand, tend to be larger at more depolarized prepulse (e.g., +60 mV), and such a tendency also remains similar at 15 °C and 25 °C. These findings are consistent with the idea that the key pathophysiological factor for the symptomatic pathogenesis is the altered temperature dependence of the kinetics in the molecular transitions more directly responsible for the genesis of resurgent currents (e.g. transitions between the open and inactivated states, see Discussion).

### Slower recovery from inactivation of the mutant than the WT Na_v_1.7 channels at 15 °C but not at 25 °C

**Figure 4:**
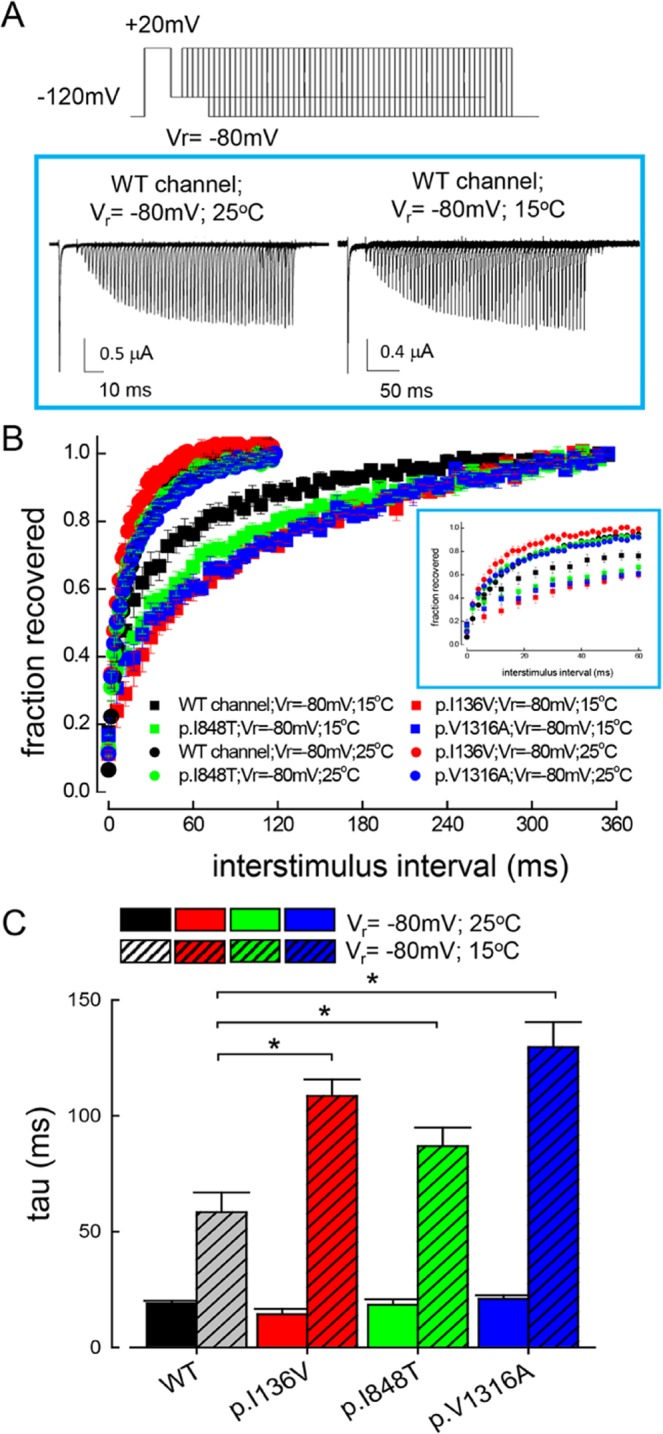
Recovery from inactivation at −80 mV in the WT, p.I136V, p.I848T, and p.V1316A mutant channels at 15 °C and 25 °C. (**A**) The cell was held at −120 mV and pulsed twice to +20 mV (each for 10 ms) every 1.5 sec, with a gradually lengthened gap between the two pulses at −80 mV (the recovery voltage, V_r_). The sweeps are arranged so that the currents in the second pulse are gradually shifted rightward as the gap was lengthened (by 0.1 ms between each sweep). (**B**) The fraction recovered is defined as the ratio between the peak current in the second pulse and that in the first pulse from the experiments in part **A** and is plotted against the duration of V_r_ to make the time course of recovery from inactivation. Note the evidently faster recovery in the WT than in the p.I136V, p.I848T, and p.V1316A mutant channels at 15 °C but not at 25 °C. Inset, first 60 ms data are redrawn with a smaller horizontal scales to demonstrate that the significantly faster recovery in the WT channel than in the mutant channels at 15 °C. (**C**) The time courses of recovery from inactivation in part **B** are fitted with a mono–exponential equation. Cumulative results of the time constants from the fits (each n = 4–5 for measurement) show that the recovery from inactivation at −80 mV is much faster at 25 °C than that at 15 °C in all channels, but is significant different between the WT and the mutant channels only at 15 °C but not at 25 °C (*p < 0.05).

The time to peak in Fig. 3 is a parameter closely related to the recovery kinetics from inactivation. Consistently, Fig. 4A,B show evident temperature–dependent changes in the kinetics of recovery from inactivation at −80 mV (a potential close to −60 mV, the voltage ranges for elicitation of resurgent currents in the previous figures). The recovery rates are similar for all channels at 25 °C. The recovery is markedly slowed at 15 °C, especially in the mutant channels (Fig. 4B,C). It is very difficult to get similar data at 40 °C because of the long–lasting duration of the experiments. Interestingly, the recovery rates are slowed to essentially the same level in the p.I136V, p.I848T, and p.V1316A mutant channels at 15 °C from the much more incongruent and faster values at 25 °C (Fig. 4B,C). On the other hand, the recovery rates are similar between the WT and the mutant channels at −120 mV either at 25 °C or 15 °C (Supplementary Data Fig. S6). The characteristic difference between the WT and the mutant channels at 15 °C but not at 25 °C is consistent with the idea that the key functional attribute responsible for the symptomatic pathogenesis of IEM lies in the direct making of resurgent currents, or more precisely, the processes involved in the recovery form inactivation of the channel (see Discussion).

### Clinical correlation between the biophysical changes in mutant channels and sensory axonal excitability of IEM

**Figure 5:**
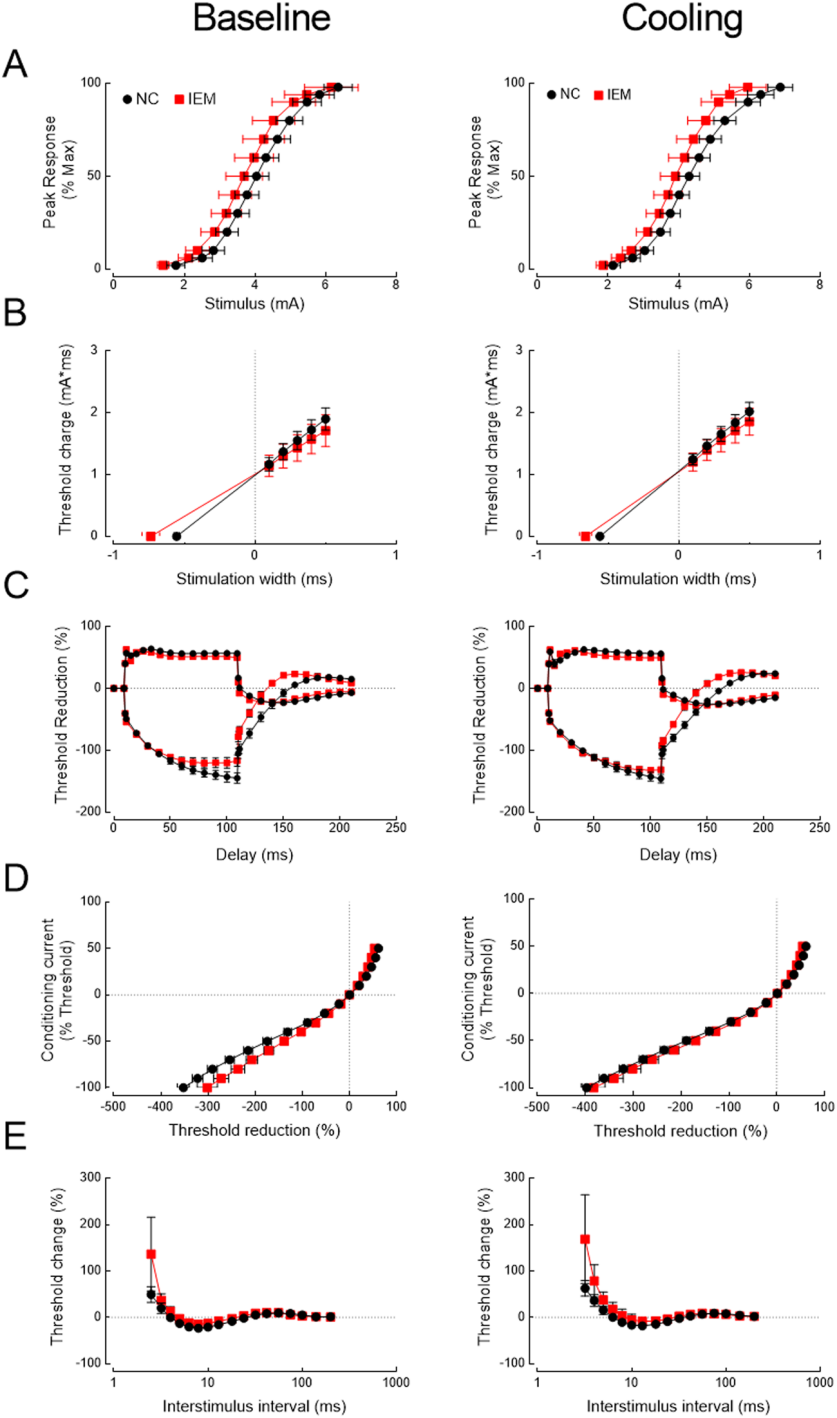
Nerve excitability test (NET) in patients with inherited erythromelalgia (IEM) and in normal controls (NC). Nerve excitability tests in participants (NC, black dots and IEM, red squares) were performed in baseline condition and after local skin cooling. The skin temperature was lowered from 36.2 ± 0.3 °C for NC (n = 10) and 36.0 ± 0.4 °C for IEM (n = 4) at baseline to 25.6 ± 1.4 °C for NC and 24.4 ± 1.2 °C for IEM after cooling, respectively. (**A**) stimulus–response (SR) curve. (**B)**. Strength–duration curve. **C**. Threshold electrotonus (TE) curve. (**D**) Current–threshold (I/V) curve. (**E**) Recovery cycle (RC).

To explore the clinical correlation of channel kinetic dysfunction, we investigated four patients (two males and two females; one female with p.I848T and the other three with p.I136V mutations) and 10 age– and gender–matched normal controls (five in each gender) with NET. The clinical profiles, medications and functional performances were shown in Supplementary Data Table 1. In the functional categories of SF–36 questionnaires, the patients reported a clear trend of limitations in physical function and in health related to pain in summer (ambient temperature 27.7–29.6 °C in Taiwan) than in winter (16.1–17.9 °C) (Supplementary Data Table 1). Consistently, the patients also reported that local cooling always relieved the pain. The NET findings are shown in Fig. 5 and Supplementary Data Table 1. There was no significant statistical difference in both resting threshold and rheobase (Fig. 5A,B) but strength–duration time constant (SDTC) is significantly increased in IEM patients (Fig. 5B). The increase appears to be smaller but remains significant with local cooling. The threshold elevation at hyperpolarizing threshold electrotonus (TE) during the late period (50–100 ms) is significantly reduced in IEM patients (Fig. 5C). Interestingly, this reduction is mostly ameliorated by local cooling (e.g., threshold reduction in both TE_h(90–100ms)_ and TE_h(99ms)_ in Table 1). The current–threshold relationship (IV) curves also show similar increase in inward rectification with hyperpolarization preconditioning in IEM patients at baseline (threshold reduction in IV_−80%_ and IV_−100%_ in Table 1). The difference of the threshold changes after preconditioned hyperpolarization, once again, becomes less prominent with cooling (Fig. 5D and Table 1). The indices of recovery cycle (RC) test demonstrated the significantly reduced superexcitability and a trend of increased refractoriness in IEM patients, especially with short (<10 ms) interpulse intervals and cooling (Fig. 5E). These findings lend a further support that there is a deranged recovery kinetics from inactivation in mutant Na_v_1.7 channels, and cooling could especially slow the recovery and increase of refractoriness in the mutant channels, resulting in an ameliorating effect on IEM symptoms (see Discussion).

**Table 1:** Temperature effect on NET indices of the median sensory axons for both normal controls (NC) and IEM patients (IEM).

	Baseline			Cooling		
	NC (n = 10)	IEM (n = 4)	p*	NC (n = 10)	IEM (n = 4)	p**
Age (year)	27.7 ± 0.7	27.3 ± 3.8	1.000	27.7 ± 0.7	27.3 ± 3.8	1.000
Temperature (°C)	36.2 ± 0.3	36 ± 0.4	0.831	25.6 ± 1.4	24.4 ± 1.2	0.887
**Strength**–**duration curve**						
Rheobase (mA)	1.81 ± 0.18	1.41 ± 0.23	0.724	1.92 ± 0.14	1.62 ± 0.2	0.525
**SDTC (ms)**	**0**.**554** ± **0**.**019**	**0**.**737** ± **0**.**063**	**0**.**020**	**0**.**558 ± 0**.**013**	**0**.**66 ± 0**.**043**	**0**.**056**
**Threshold Electrotonus (TE)**						
TE_d(10_–_20ms)_ (%)	60.1 ± 2.1	59.1 ± 2.2	0.525	51.1 ± 2.2	57.4 ± 1.6	0.179
TE_d(90_–_100ms)_ (%)	56.8 ± 2.2	50.6 ± 3.2	0.179	56.2 ± 2	49.5 ± 1.8	0.056
TE_h(10_–_20ms)_ (%)	−82.3 ± 3.2	−83.8 ± 3.1	0.621	−79 ± 3.1	−82.6 ± 4	0.437
**TE**_**h(90**_ **–**_**100ms)**_ **(%)**	**−143**.**9 ± 8**.**2**	**−118**.**6 ± 8**.**8**	**0**.**104**	**−144**.**1 ± 7**	**−131**.**8 ± 3**.**6**	**0**.**358**
**TE**_**h99ms**_ **(%)**	**−144**.**8 ± 8**.**2**	**−116**.**8 ± 9**.**2**	**0**.**138**	**−145**.**7 ± 6**.**9**	**−131**.**4 ± 3**.**4**	**0**.**358**
TE_h,slope (101_–_140)_ (%/ms)	2.32 ± 0.13	2.32 ± 0.17	1.000	2.05 ± 0.1	2.42 ± 0.11	0.104
TE_h,overshoot_ (%)	19.9 ± 1.8	23.6 ± 3.1	0.289	21.3 ± 2.1	25.7 ± 2.4	0.289
**Recovery cycle**						
Relative refractory period (ms)	3.97 ± 0.37	4.91 ± 0.47	0.138	5.91 ± 0.79	6.61 ± 1.17	0.933
Refractoriness (%)	49.3 ± 17.1	136.3 ± 79.4	0.138	113.5 ± 30.9	98.7 ± 66.3	0.915
**Superexcitability (%)**	**−23**.**7 ± 1**.**9**	**−15**.**5 ± 1**.**6**	**0**.**040**	**−18**.**7 ± 2**.**4**	**−11**.**8 ± 5**.**6**	**0**.**358**
Subexcitability (%)	10 ± 0.7	10.4 ± 0.5	0.438	8.2 ± 0.7	9.1 ± 0.5	0.437
**Current–threshold (I/V) curve**						
Hyperpolarizing IV slope	0.348 ± 0.031	0.324 ± 0.042	0.832	0.283 ± 0.033	0.251 ± 0.021	0.944
**IV**–_**80%**_**(%)**	**−290**.**7 ± 10**.**2**	**−236**.**5 ± 14**.**2**	**0**.**020**	**−320**.**6 ± 8**.**6**	**−301 ± 17**.**1**	**0**.**437**
**IV**–_**100%**_**(%)**	**−352**.**2 ± 12**.**8**	**−301**.**5 ± 21**.**3**	**0**.**077**	**−397**.**2 ± 11**	**−382**.**6 ± 23**.**6**	**0**.**525**

This table made by conducting Mann–Whitney test, and the distribution was presented by mean ± S.E.M.

*Compared to NC at normal temperature.

**Compared to NC at cooling.

## Discussion

Our current works focus on the molecular pathogenesis of heat–exacerbated pain in IEM. Hyperpolarization shift of activation curve and increased window current have long been considered as key contributors of neural hyperexcitability in IEM^3^. However, most biophysical properties of these mutants have been obtained in room temperature^17–19^, and the temperature effects on these properties have been incongruent across different mutants^7,11^. To prevent overemphasis on properties in a particular mutant, we carefully seek common properties in three mutants, p.I136V, p.I848T and p.V1316A, which are in different domains of Na_v_1.7. The major findings are summarized in Table 2.

**Table 2:** Biophysical properties in WT and IEM–related mutant, p.I136V, p.I848T, and p.1316 A, Na_v_1.7 channels in three different temperatures.

Mutation (position)	Tested temperature (°C)	Activation		Fast inactivation				Area of window current	
		V_h_ (mV)	k	V_h_ (mV)	k	τ, inactivation	τ, recovery from −80 mV (ms)		
WT	25	−21.8 ± 1.5	8.3 ± 0.43	−85.7 ± 2.1	−12.5 ± 0.33		18.98 ± 0.34	1.13 ± 0.001	
	40	−20.4 ± 1. 1	10.6 ± 0.32	−83.8 ± 1.9	−14.3 ± 0.34			1.51 ± 0.001	
	15	−27.5 ± 1.2	6.1 ± 0.27	−76.9 ± 1.7	−13.7 ± 0.42		58.34 ± 0.23	1.78 ± 0.002	
p.I136V	25	−27.5 ± 1.4	7.7 ± 0.23	−80.5 ± 1.2	−12.8 ± 0.21	↑	14.34	1.53 ± 0.001	
(VSD)	40	−15.2 ± 1.2	7.5 ± 0.42	−67.0 ± 1.1	−13.8 ± 0.21	n.c.		1.78 ± 0.0001	
	15	−36.8 ± 1.2	7.02 ± 0.34	−64.5±1.2	−13.2 ± 0.43	↑	108.52	3.86 ± 0.0001	
p.I848T	25	−32.6 ± 1.2	7.6 ± 0.23	−73.5 ± 1.3	−12.5 ± 0.37	↑	18.39	2.38 ± 0.001	
(S4–S5)	40	−18.5 ± 1.2	8.8 ± 0.23	−60.6 ± 1.4	−14.3 ± 0.12	n.c.		3.52 ± 0.001	
	15	−41.5 ± 1.4	6.8 ± 0.21	−63.6 ± 1.2	−10.6 ± 0.24	↑	86.85	3.12 ± 0.0001	
p.V1316A	25	−34.4 ± 1.3	8.7 ± 0.34	−77.1 ± 0.9	−11.9 ± 0.12	↑	20.93	2.0 ± 0.001	
(S4–S5)	40	−41.7 ± 1.4	9.1 ± 0.32	**−86**.**7 ± 0**.**8**	−13.1 ± 0.32	n.c.		2.76 ± 0.0001	
	15	−34.2 ± 1.5	7.4 ± 0.27	−68.7 ± 0.8	−12.3 ± 0.33	↑	129.61	4.57 ± 0.0001	
Ref.		Figs 1 and 2, Fig. S1		Figs 1 and 2, Fig. S1		Fig. S2	Fig. 4; Fig. S6	Fig. 2, Fig. S1	
**Mutation (position)**	**Tested temperature (°C)**	**Resurgent current**							
		**Peak (to I**_**transient**_ **)**	**Ratio of I**_**resurgent**_ **/I**_**transient**_ **to WT at the same temperature**	**Time to peak**	**τ, decay**	**V**_**h**_ **, activation (mV)**	**k, activation**	**τ, decay after depolarization at 60 mV (ms)**	**f**_**0**_ **, decay after depolarization at 60 mV**
WT	25	0.071 ± 0.003				44.5 ± 2.5	28.6 ± 1.5	64.2 ± 1.9	0.4 ± 0.03
	40	0.05 ± 0.01				44.7 ± 1.9	19.1 ± 1.5	n.d.	n.d.
	15	0.42 ± 0.03				44.8 ± 2.2	29.3 ± 1.3	21.4 ± 2.0	0.4 ± 0.02
I136V	25	0.12 ± 0.01	1.69 (0.12/0.071)	↑	↑	45.8 ± 2.2	29.0 ± 1.8	70.4 ± 1.6	0.45 ± 0.03
(VSD)	40	0.13 ± 0.01	2.6 (0.13/0.05)	↑↑	↑	44.7 ± 2.9	19.1 ± 1.9	n.d.	n.d.
	15	0.48 ± 0.02	1.14 (0.48/0.42)	↑	↑	37.6 ± 2.1	17.8 ± 1.2	29.4 ± 2.2	0.36 ± 0.01
I848T	25	0.19 ± 0.01	2.67 (0.19/0.071)	↑	↑	58.8 ± 2.17	30.3 ± 1.13	69.8 ± 1.5	0.55 ± 0.03
(S4–S5)	40	0.22 ± 0.01	4.4 (0.22/0.05)	↑↑	↑	49.1 ± 2.1	21.8 ± 1.2	n.d.	n.d.
	15	0.50 ± 0.05	1.19 (0.5/0.42)	↑	↑	34.6 ± 1.9	31.1 ± 1.4	53.6 ± 2.7	0.5 ± 0.03
V1316A	25	0.12 ± 0.01	1.5 (0.12/0.071)	↑	↑	45.8 ± 2.5	30.3 ± 1.9	86.0 ± 1.7	0.40 ± 0.05
(S4–S5)	40	0.17 ± 0.02	3.4 (0.17/0.05)	↑↑	↑	40.8 ± 2.3	21.7 ± 2.0	n.d.	n.d.
	15	0.34 ± 0.02	0.81 (0.34/0.42)	↑	↑	38.8 ± 2.0	36.7 ± 2.3	41.2 ± 2.0	0.40 ± 0.05
Ref.		Fig. 3	Fig. 3; Fig. S3	Fig. 3	Fig. 3	Fig. S4	Fig. S4	Fig. S5	Fig. S5

Activation or inactivation curves are shown using the Boltzmann function: 1/[1 + exp((V_h_ − V)/*k*)]. Arrows in the table are denoted the comparison between mutant and WT channel at the same temperature. (VSD = voltage sensing domain; WT = wildtype).

n.c.: not changed.; n.d.: not performed, the distribution was presented by mean ± S.E.M.

In our data, heating does not cause further hyperpolarizing shift in all mutants, and even ameliorated the hyperpolarizing shift of activation curve in p.I136V and p. I848T (Fig. 2), while temperature change induces a bimodal increase of window current at higher and lower temperatures (Fig. 2). These *in vitro* data indicate that the steady–state sustained window currents are probably not decisive for the genesis of rapid repetitive discharges and thus the clinical symptoms upon heating. In contrast, the increase in relative resurgent currents at 40 °C is chiefly due to the decreased peak currents because of the accelerated inactivation. Although the absolute peak seems to be reduced at 40 °C than at 25 °C and 15 °C, the excitability of the cell is increased rather than decreased at 40 °C because the kinetics of the Na^+^ currents are accelerated (Fig. 3H–J). In other words, the more rapid onset of the transient as well as resurgent Na^+^ currents (and thus the shorter time to peak) would shorten the time of bringing the membrane potential to the threshold for firing an action potential. The larger relative resurgent currents (relative to the transient currents even it is chiefly due to the smaller transient currents), with a shortened time to peak, therefore should still make a valid signal of increased excitability. On the other hand, the activation curve of resurgent currents and the kinetics of genesis of the inactivated states during the depolarization prepulse in different temperatures are rather similar for the WT and mutant channels (Figs S4 and S5). These findings indicate that the altered kinetics (or more precisely, altered temperature dependence of the kinetics) of the molecular transitions directly related to the genesis of resurgent current are the key factor contributing to the pathogenesis of heat–induced pain in IEM. The prominent increase of relative resurgent currents at 40 °C, based on a slower dissipation and/or faster genesis of the resurgent currents in the mutant channels, would acceleratedly supply the resurgent Na^+^ currents at higher temperatures and thus aggravate clinical symptoms with heating.

We also explored the biophysical abnormalities with clinical electrophysiological test. Despite of the small number of cases, the NET study in IEM patients consistently shows decrease in the threshold elevation at the hyperpolarizing TE at 50–100 ms (TE_h(50–100ms)_), increase of inward rectification in the current–voltage (I/V) curve, and increase of refractoriness at relatively short interpulse interval at 2–6 ms during RC (Fig. 5 and Table 2; c.f.^15^). Recently, the microneurography study on the C–fiber recovery cycle shows a significant increase of refractoriness in the mechanosensitive and sympathetic fibers from an IEM patient with p.I848T mutation, which is in line with our NET findings^13^. On the other hand, there is no significant difference in the stimulus–response curve, resting threshold, or the rheobase by temperature changes. These results show the essentially normal axonal excitability in IEM if assessed by single (or unpreconditioned) stimulus, but abnormalities in those NET indices obtained with preceding or preconditioning pulses. These findings could implicate abnormal gating kinetics relevant to inactivation or recovery from inactivation in the mutant Na_v_1.7 channels. Nevertheless, the case number is small in our current study. Further investigation with a bigger cohort may provide more definitive conclusions.

**Figure 6:**
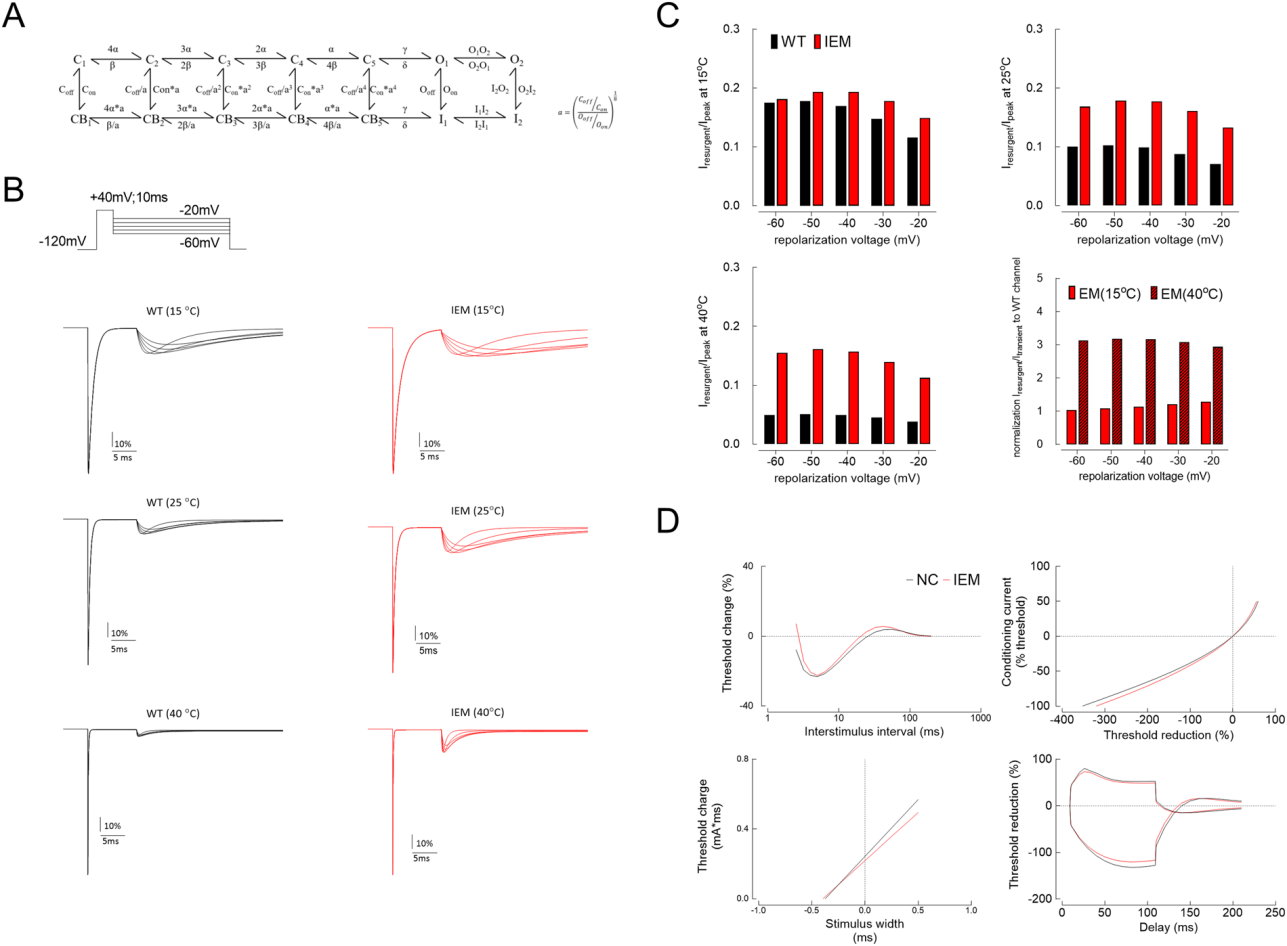
Computational simulation of resurgent Na^+^ current and corresponding excitability changes. (**A**) The gating scheme of wild–type (WT) and inherited erythromelalgia (IEM) mutant channels are shown. C, O, and I denote closed, open, and inactivated state, respectively. Note that there is a second open and corresponding inactivated states (O_2_ and I_2_) which are responsible for the genesis of resurgent currents^13^. The detailed transition rates are listed in Table 3. (**B**) The simulated currents of WT and IEM mutant channels at 15 °C, 25 °C, and 40 °C based on the same stimulation protocols as Fig. 3. (**C**) The peak of simulated resurgent Na^+^ current from part **B** based on the same experimental measurement protocols in Fig. 3. (**D**) The simulated RC, IV, TC and TE curves of WT and IEM–causing mutant channels are modelled with the kinetic parameters listed in part **A** and reduction in pump current from 30 to 15 pA (because of the presumable reduction in local perfusion, see Discussion). Simulation shows the increased refractoriness, reduced superexcitability, and increased SDTC in IEM. The threshold elevation at 99 ms of hyperpolarizing TE is also reduced, and IV curve shows more inward rectification in IEM. All these findings are consistent with the experimental findings in Fig. 5.

Albeit the IEM–causing mutations are scattered in different domains of the α–subunit of Na_v_1.7 channel, the clinical features are rather similar among IEM patients and thus indicate a relatively common functional disturbance among the mutant channels. As discussed in the previous section, the temperature–dependent changes in the resurgent current are similar in different mutant channels and show a tendency well consistent with the clinical features. Although it was argued that IEM–causing mutations (p.I848T and p.L858H in the study) did not affect resurgent Na^+^ current^16^, our previous study has shown unambiguous changes in resurgent Na^+^ current of p.V1316A mutant channel, a mutation causing prototypical IEM^14^. The discrepancy is probably partly ascribable to the different pulse protocols. The prepulse for the elicitation of the transient currents and subsequent resurgent pulse was set to +20 mV for 20 ms^16^. However, we have shown that the resurgent Na^+^ currents actually get smaller with longer prepulses, so that there could be a marked decrease of the currents with a +20 mV × 20 ms pulse^14^). We therefore chose a stronger and shorter prepulse in our studies (e.g. +40 mV for 10 ms) to check the resurgent currents in a more complete fashion. The stronger temperature dependence in both macroscopic inactivation rate and rate of recovery from inactivation at −80 mV (Fig. 4) in the mutant channels also directly demonstrate the altered channel inactivation as well as the recovery process from inactivation. All these findings on the temperature–dependent molecular gating behavior could be well simulated with a gating scheme (Fig. 6A,B; Supplementary Data Fig. S7), in which the major difference between WT and the mutant channels is in the Q_10_ of the O_2_ to I_2_ transitions (~2 and ~3 for the WT and mutant channels, respectively; Table 3). In other words, the overall recovery kinetics from inactivation are very much temperature–dependent at this temperature range in the mutant channels. Because the overall recovery from inactivation is composed of the recovery via both silent and resurgent routes, it is plausible that at a certain low temperature (e.g. 15 °C), the overall recovery is still slower in the mutant channel but the time to peak is already quite similar between the WT and mutant channels. In contrast, at a higher temperature (e.g. 25 °C), the overall recovery is comparable in the WT and mutant channels, but the time to peak now tends to be shorter in the mutant channels. The temperature–dependent changes in resurgent currents, which are generated during the recovery of the inactivated sodium channel from a previous pulse, may well affect the timing of the genesis of the following discharge and therefore explain the occurrence of paroxysms of repetitive or burst discharges with episodic symptomatic aggravation of IEM (see below).

**Table 3:** The kinetic parameters for the wild type (WT) and IEM channel models.

	WT			IEM		
	*k*_0_ (ms^−1^, at 25 °C)	*k*_1_ (mV)	Q_10_	*k*_0_ (ms^−1^, at 25 °C)	*k*_1_ (mV)	Q_10_
α	**100**	0.08	2.1	**500**	0.08	2.1
β	**0.55**	−0.01	2.1	**4**	−0.01	2.1
γ	40	0.01	2.1	40	0.01	2.1
δ	2	−0.01	2.1	2	−0.01	2.1
C_off_	0.5		3.2	0.5		3.2
C_on_	0.1		3.2	0.1		3.2
O_off_	0.004		3.2	0.004		3.2
O_on_	2.5		3.2	2.5		3.2
O_1_O_2_	0.12	0.057	**2**	0.12	0.057	**2.6**
O_2_O_1_	0.001	−0.095	**2**	0.001	−0.095	**2.6**
O_2_I_2_	**0.5**	0.04	**2**	**0.3**	0.04	**3.3**
I_2_O_2_	**0.06**	−0.03	**2**	**0.07**	−0.03	**3.3**
I_1_I_2_	**0.002**	0.01	2.6	**0.001**	0.01	2.6
I_2_I_1_	**0.2**	−0.038	2.6	**0.12**	−0.038	2.6

All transition rates are expressed as *k*_0_·*e*^k1*E^·Q10 ^*(T*–*T0)/10*^, where *k*_0_ is the transition rate at 0 mV, *k*_1_ is the voltage dependence constant in mV^−1^, *E* is membrane potential in mV, Q_10_ is the temperature coefficient, and T is temperature in °C (with T_0_ at 25 °C). The basic kinetic parameters at room temperature are the same as those reported previously^21^, with slight changes in *k*_1_ of *δ* and *k*_0_ of I_2_O_2_ and O_2_I_2_ of the WT and IEM channels for better simulation of all of the findings in different temperatures. The main differences between the kinetics of WT and IEM channels are in *k*_0_ of α, β, O_2_I_2_, I_2_O_2_, I_1_I_2_ and I_2_I_1_ and the temperature dependence of transition between states O_2_ and I_2_ (bold letters).

**Figure 7:**
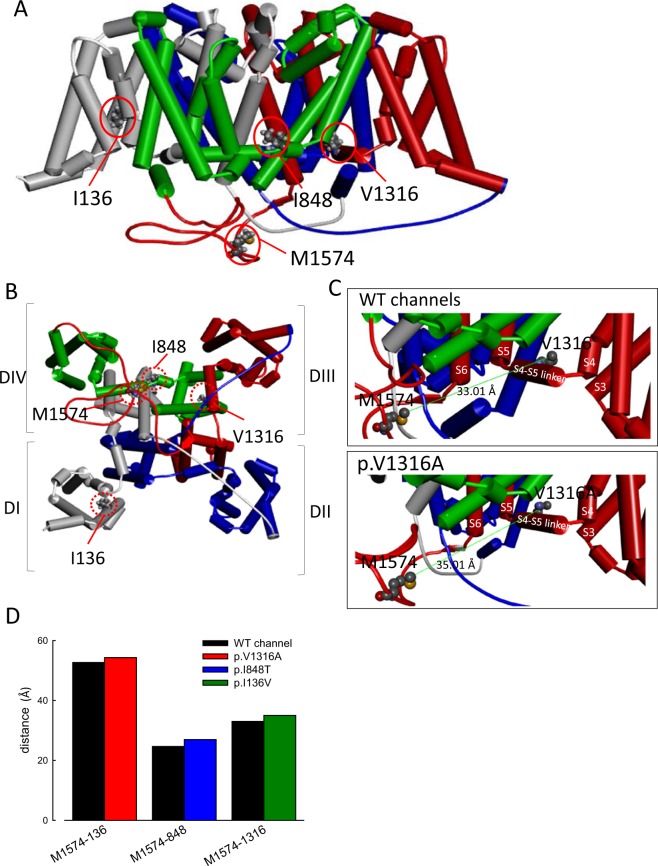
Homology modeling of the WT and mutant human Na_v_1.7 channels. (**A**) The homology model was constructed according to the X-ray crystal structures of human voltage-gated Na^+^ channel (Na_v_1.7) using the Discovery Studio 2018 software (see Material and Methods for detail). A side view of the homology model of WT human Na_v_1.7 channel shows the transmembrane regions of the four domains. Domains I, II, III, and IV are white, blue, red, and green, respectively. The side chains of I136, I848, and V1316 in domains I, II, and III, respectively, and of M1574 in the “IFM” inactivation motif are highlighted with the ball and stick model. (**B**) A regional view of the homology model of the WT human Na_v_1.7 channel from the intracellular side of the pore. (**C**) The side chains of V1316 in the S4-S5 linker in domain III and in M1574 in the domain III-IV linker are indicated with the CPK model of different colors, showing larger tip-to-tip distance (~35.01 Å vs. ~33.01 Å) in the V1316A mutant than in the WT channel. (**D**) A summary plot of the side chain tip-to-tip distances between residues I136 (or p.I136V), I848 (or p.I848T), V1316 (or p.V1316A), and M1574 in the homology model of human Na_v_1.7 channel. Note the increased distances in all of the mutant if compared with that in the WT channels.

A characteristic and congruent change among different mutations is the paradoxically increase of resurgent current at higher temperature. Although the resurgent current at lower temperature (15 °C) is increased in both WT and mutant channels, the difference in between is much reduced as compared to that found at 40 °C. Both the experimental data and the computer simulation strongly suggest that the altered transitional rates between O_2_ and I_2_ states (Fig. 6B and Table 3) play a major role in the genesis of the deranged resurgent currents in IEM. Interestingly, the absolute differences in these transitional rates could be minimal or even negligible between WT and mutant channels at lower temperatures (such as 15 °C). However, if the Q_10_ of the designated transitions is changed from ~2 (in the WT) to ~3 (in the mutant channels, Fig. 6 and Table 3), a marked difference of resurgent currents occurs at 40 °C. Thermodynamically speaking, such a change in Q_10_ signals an activation energy change from 1.1 to 0.69 RT when the temperature is elevated by 10 °C. Also, this energy difference is most likely due to the change in entropy to suffice the altered temperature dependence or Q_10_. It is consistent with the view that the genesis of resurgent currents involves large–scale conformational changes of the channel protein, most likely involving the changes in multiple hydrophobic bonding, rather than a simple competition between two pore blockers (e.g. the inactivating peptide and the “resurgent” particle)^14^, which also explains why the point mutations dispersed over different domains in the channel could cause the similar gating changes as well as the similar temperature-dependent symptomatology. With homology modeling of the human Nav1.7 channel (Fig. 7), it is also noted that the p.I136V, p.I848T, and, p.V1316A mutations all increase the inter-residue distances between these mutant residues and the methionine (M1574) in the “IFM” motif which is critical for channel inactivation^20–25^. The conformational changes associated with p.I136V, p.I848T, and, p.V1316A mutations may thus involve imperative machineries of channel inactivation, and alter the processes of recovery from inactivation as well as genesis of resurgent currents. Given the possibility that the key factor for the symptomatic pathogenesis of IEM is the abnormally enhanced and “accelerated” resurgent currents in the mutant Na_v_1.7 channels, the search for an effective gating modifier in this regard may be a rewarding and fundamental approach for a successful pharmacotherapy of this excruciating disorder.

## Materials and Methods

### Mutagenesis and expression of DNA constructs of Na_v_1.7 channel

The full–length cDNA of WT human Na_v_1.7 channel (SCN9A gene) was purchased from OriGene Technologist (Rockville, MD, USA) and subcloned into pTracer–EF/V5–His vector^14^. The sequence encoding green fluorescence protein (GFP) was cloned by a separate promoter in the pTracer–EF/V5–His vector. For mutagenesis, cDNA of mutant channels (p.I136V, p.I848T, and p.V1316A) was created using QuikChange Site–Directed Mutagenesis System kits (Stratagene, La Jolla, CA, USA). The identity of each mutant channel was confirmed by automatic DNA sequencing (3730xl DNA Analyzer; Applied Biosystems, Foster, CA, USA) in the third common laboratory of National Taiwan University Hospital.

### Preparation of CHO−K1 cell line for DNA transfection

Chinese hamster ovary (CHO−K1) cells, obtained from the Food Industry Research and Development Institute (Hsinchu, Taiwan), were maintained under humidified conditions of 5% CO_2_/95% O_2_ at 37 °C in F12−K medium (Thermo Fisher Scientific Inc., USA) supplemented with 10% fetal bovine serum (FBS; Thermo Fisher Scientific Inc., USA) and 1% penicillin–streptomycin–ampicillin antibiotics (PSA; Thermo Fisher Scientific Inc., USA)^14^. A total of 0.5–1.0 × 10^6^ CHO−K1 cells were seeded onto a 3.5 cm culture dish (Greenpia Technology Inc., South Korea) the day before DNA transfection using Lipofectamine™ 3000 (Thermo Fisher Scientific Inc., USA). For electrophysiological recordings, the CHO−K1 cells were suspended with 0.5 mg/ml trypsin (Sigma Chemical Co., St Louis, MO, USA) and plated on glass cover–slips under conditions of 5% CO_2_/95% O_2_ at 37 °C for 10 min before wash–off of trypsin with phosphate–buffered saline.

### Electrophysiological recording of CHO−K1 cells expressing DNA constructs

The electrophysiological recording of CHO−K1 cells was performed 48–72 h after DNA transfection. Whole–cell patch clamp recordings for the cell expressing WT and different mutant channels were carried out using with an Axopatch 200A amplifier and pClamp 6.0 software (Axon Instruments, Inc., Sunnyvale, CA, USA). The recorded currents were digitized at a rate of 50 kHz after passing through a low–pass Bessel filter at 10 kHz through a Digidata 1200A interface (Axon Instruments, Inc., Sunnyvale, CA, USA). A pipette with an internal tip diameter of 0.5–1.0 μm was pulled from borosilicate micropipettes (Sutter Instrument, Inc., CA, USA) using a puller (Zeitz Instrument Inc., Martinsried, Germany). The pipette resistance was 1.5–2.5 mΩ when filled with an intracellular solution containing 75 mM CsCl, 75 mM CsF, 5 mM HEPES, 2 mM CaCl_2_, and 2.5 mM EGTA, titrated by 1.0 M CsOH to pH 7.4. The whole–cell configuration was formed in an extracellular solution 145 mM NaCl, 10 mM HEPES, 2 mM CaCl_2_, and 2.5 mM MgCl_2_ titrated by 1.0 M NaOH to pH 7.4. The cell was moved in front of a linear array of pipes (Drummond Scientific Co., Broomall, USA) emitting different extracellular solution. Unless otherwise specified, a short peptide of Na_v_β4 peptide (KKLITFILKKTREK–OH, 0.1 mM) corresponding to the C–terminal half of the full sequence of the Na_v_β4 subunit, was added to the intracellular solution and allowed to diffuse into the CHO–K1 cells until response were stable for about 3 min before every experiment. The tetrodotoxin–sensitive (TTX–s) and resurgent currents were determined by subtraction of the currents following 1.0 μM TTX (Tocris, Cookson, Langford, UK).

### Effect temperature on the WT and different mutant channel

To evaluate the effects of temperature on the transient and resurgent currents of WT and mutant channels, we exercised temperature control at 15 °C, 25 °C, and 40 °C using an automatic temperature controller (TC–324B and TC–324C; Warner Instruments Corporation, USA). The flow rate of external solution was 0.3 ml/min and the volume of the recording chamber was approximately 5 ml. The recording chamber was maintained at 15 °C, 25 °C, and 40 °C for at least 3 min to ensure homogeneity of the temperature control, which was also confirmed by direct measurement of the external solutions emitted from pipes with appropriate sensors.

### Construction of the activation and inactivation curves for WT and mutant Na_v_1.7 channels

To determine the current–voltage (I–V) relationship, cells were held at −120 mV and subjected to various test pulses increasing in 5 mV increments from −160 to +40 mV for 100 ms. The inter-sweep interval was 1.5 sec. The maximal amplitude of sodium inward currents was plotted as a function of test voltage to generate the I–V plot, including a regression line between + 10 and +40 mV. The reversal potential of sodium was determined by extrapolating the regression line to the transverse axis, and peak sodium conductance (G_max_) was obtained from the slope of this line. Normalized sodium conductance was defined as I_peak_/[(V − V_Na_^+^) × G_max_], where I_peak_ and V denote peak sodium amplitude and test voltage during different depolarizations, respectively. The normalized sodium conductance was plotted against the membrane voltage, and then fitted with a Boltzmann function: G/G_max_ = 1/[1 + exp(V_h_ − V)/k)], where k is the slope factor, G_max_ is peak Na^+^ conductance, V_h_ is the potential at half–maximal activation, and V is the membrane voltage, to make the activation curve. For the steady–state inactivation curve of sodium currents, the peak amplitude at a +10 mV test pulse was documented after a 100 ms prepulse at different membrane voltages from a holding potential of −120 mV^14^. The peak current at the test pulse was normalized to the maximal one (I/I_max_) and plotted against the prepulse membrane voltage to construct the inactivation curve. The inactivation curve was then fitted with a Boltzmann function: I/I_max_ = 1/[1 + exp(V − V_h_)/k)], where k is the slope factor, I_max_ is maximal peak current, V_h_ is the potential at half–maximal inactivation, and V is the prepulse membrane voltage^14,26–31^. The fittings to the activation and inactivation experimental data sets were performed using Origin 6.0 software (Microcal Software, Inc., Northampton, MA, USA). The area of windows currents was based on the activation and inactivation curves in the WT and different mutant channels, and derived using the Sigmaplot 10.0 (Systat Software. Inc.).

### Clinical profile and Nerve excitability test (NET)

All patients and healthy controls were recruited from the Department of Neurology, National Taiwan University Hospital, Taipei, Taiwan, and had provided the written informed consent. All procedures were approved by the Research Ethics Committee of National Taiwan University Hospital (201610049RINB), Taipei, Taiwan. We have followed approved procedures and ethical guidelines by declaration of Helsinki. Patients who harbor a Na_v_1.7 channel (SCN9A gene) mutation and meet the following clinical criteria were recruited: (1) redness, warmth, and pain in both legs; (2) symptomatic exacerbation by warmth and exercise; (3) relief of symptoms by local cooling; (4) lacking of a secondary cause such as myeloproliferative disease, diabetes, infection, connective tissue diseases, or paraneoplastic syndrome^32,33^. The patients were asked to document the SF–36 Health Survey Questionnaire regarding the clinical conditions both in summer (July) and in winter (January) seasons^34^. All subjects received NET in the sensory axons of the left median nerve (detailed procedures in previous studies^35^). In brief, the stimulation was delivered by an isolated linear bipolar stimulator, DS5 (Digitimer, Welwyn Garden City UK), which was triggered by a computerized software QTRACS (Institute of Neurology, London, UK). According to the TRONDNF protocol (Version 18/8/2008, copyright, Prof. Hugh Bostock, Institute of Neurology, London), each session included five stimulation protocols, including stimulus–response (SR) curve, strength–duration (SD) relationship, threshold electrotonus (TE), current–threshold (I/V) relationship, and recovery cycle (RC)^36,37^. The target threshold was set at 40% of the amplitude of maximal sensory action potential (SAP) amplitude. Although the feet are usually more affected by the symptoms of pain, local edema and ulcers commonly existed. We therefore chose upper limb for examination. All NET procedures were repeated twice at normal body (~35 °C) and at a cooled skin temperature (10 °C lower than patient’s baseline by local ice packing). Skin temperature was frequently monitored during the examination, and heating with a warm light would be applied to restore the designated temperature if necessary. For analysis, the results of indices were extracted by QTRACP program (Institute of Neurology, London). At RC test, the refractoriness is defined by threshold change measured at an inter–stimulus interval of 2.5 ms for baseline, but of 3.2 ms for cooling because some subjects showed absolute inexcitability at 2.5 ms.

### Numerical simulation of resurgent currents and the results of NET

The Markov model for resurgent sodium current was adopted and modified from our previous study^14^. Q–matrix method was employed to accelerate the computation in simulating voltage–clamp study and NET. Parameters such as activation– and inactivation–curves, peak resurgent currents, time to peak resurgent current, resurgent activation curves and decay of resurgent current, were adjusted to fit the experimental results. For simulation of voltage–clamp recordings, the ionic currents were assumed to have Ohmic relationship, and the reversal potential was set to +100 mV. The derived model from voltage–clamp study was then used to simulate the findings from NET. A mathematical model for human myelinated axon was modified from Bostock’s model, with modifications in the current conductance, pump current and voltage dependence of HCN currents. Because the Hodgkin–Huxley type kinetics for sodium current in the original Bostock’s model has disregarded the occurrence of resurgent currents, we applied the Markov model of sodium channel to simulate resurgent currents^38,39^. Specifically, twenty–seven percent of transient sodium conductance was replaced by Markov model for resurgent sodium current, according to the percentage of Na_v_1.7 expressed on peripheral axons^40^. The detailed equations of mathematical model for human myelinated axon were listed in supplementary data. The time–step was set to 10^−3^ ms. The computation was performed by MATLAB R2015 suite (The MathWorks, Inc. US).

### Homology modeling of the human Na_v_1.7 channel

A homology model of the human Na_v_1.7 channel encoded by the *SCN9A* gene was built according to coordinates acquired from the X-ray crystal structure data of the human voltage-gated Na^+^ channel Na_v_1.7 (human Na_v_1.7; PDB code: 6J8H)^41^. Homology modeling was performed in a manner similar to that described in previous studies^14,42–45^. The amino acid sequence of the human Na_v_1.7 channel was obtained from the UniProt database (Q15858)^14^. The complete sequences of the three interdomain linkers of the human Na_v_1.7 channel (DI-DII linker, C740–P789; DII-DIII linker, M1190–Y1239; and DIII-DIV linker, D1447–V1519) were inserted into the corresponding positions between different domains (DI–DIV) according to a template of the structure of the human Na_v_1.7 channel^14^. Aligned sequences were then processed using the Discovery Studio 2018 client software to generate the secondary structure and to assign the relative positions of the human Na_v_1.7 channel^14,42–45^. The conformation with the lowest free energy potential determined using the Discrete Optimized Protein Energy score was chosen for further analysis^14,42–45^.

### Experimental design and statistical analysis

Data were presented as mean ± standard error of mean (S.E.M.) with sample size, n, indicating the number of cells from which each dataset was collected. Error bars represent S.E.M. The statistical significances were assessed using one–way ANOVA (Figs 1, 2, and Supplementary Data Figs S1 and S3), Kruskal–Wallis test (Fig. 3), and two–way ANOVA (Fig. 4, and Supplementary data Fig. S6), the Mann–Whitney U test for two independent samples (Fig. 5), with a statistically significant level of p < 0.05. Data analyses were performed using the SPSS 20.0 Statistics (New York; United states).

## Supplementary information

SUPPLEMENTARY INFO

## Data Availability

All data generated or analyzed during this study are included in this published article and its Supplementary Information files.
