# Supplementary material for: Anomalous enhancement of resurgent Na+ currents at high temperatures by SCN9A mutations underlies the episodic heat-enhanced pain in inherited erythromelalgia
Source: Sci Rep. 2019 Aug 22;9:12251. doi: 10.1038/s41598-019-48672-6 (PMC6706385; doi:10.1038/s41598-019-48672-6)
Supplement: Supplementary file 1 — SUPPLEMENTARY INFO [file 41598_2019_48672_MOESM1_ESM.docx]

**Supplementary data**

**Anomalous enhancement of resurgent Na^+^ currents at high temperatures by SCN9A mutations underlies the episodic heat-enhanced pain in inherited erythromelalgia.**

Chiung–Wei Huang, Ph.D. ^5, #^, Hsing–Jung Lai, M.D. ^1, 2, 3, #^, Po–Yuan Huang, Ph.D. ^3^, Ming–Jen Lee, M.D., Ph.D. ^3, 4, *^, Chung–Chin Kuo, M.D., Ph.D. ^1, 3, *^

1. Department of Physiology, National Taiwan University College of Medicine, Taipei, Taiwan.
2. Department of Neurology, National Taiwan University Hospital Jinshan Branch, New Taipei City, Taiwan.
3. Department of Neurology, National Taiwan University Hospital, Taipei, Taiwan.
4. Department of Medical Genetics, National Taiwan University Hospital, Taipei, Taiwan.
5. Department of Physiology, Kaohsiung Medical University, Kaohsiung, Taiwan

# These authors contribute equally

**Extended Table 1. Clinical profile of patients with inherited erythromelalgia (IEM)**

|  | Patient 1 | Patient 2 | Patient 3 | Patient 4 |  |
| --- | --- | --- | --- | --- | --- |
| Age | 19 | 23 | 31 | 36 |  |
| Gender | F | M | M | F |  |
| Genotype | p.I136V | p.I136V | p.I136V | p.I848T |  |
| Family history | No | Yes | Yes | No |  |
| Presenting symptoms | Pain, erythema, ulcer | Pain, erythema, ulcer | Pain, erythema | Pain, erythema |  |
| Symptomatic limbs | LE | LE | LE | LE |  |
| Medications at examination (/day) | None | MX 200mg, CBZ 600mg | None | MX 100mg, CBZ 600mg |  |
| SF–36**  (%, Summer/Winter) |  |  |  |  | p* |
| General health | 35/55 | 75/75 | 50/50 | 65/75 | 0.18 |
| Physical functioning | 35/80 | 55/70 | 50/55 | 50/80 | 0.07 |
| Role limitations  due to physical health | 0/0 | 25/100 | 50/50 | 0/100 | 0.10 |
| Role limitations  due to emotional problems | 0/100 | 0/100 | 66.7/66.7 | 66.7/100 | 0.10 |
| Energy/fatigue | 35/65 | 40/80 | 50/50 | 25/80 | 0.10 |
| Emotional well–being | 24/80 | 68/72 | 60/60 | 56/76 | 0.11 |
| Social functioning | 12.5/62.5 | 37.5/75 | 62.5/62.5 | 37.5/75 | 0.18 |
| Pain | 0/90 | 22.5/67.5 | 47.5/47.5 | 22.5/67.5 | 0.10 |

MX: mexiletine; CBZ: carbamazepine; LE: lower extremities

*: comparison between conditions in winter and summer, Wilcoxon signed–rank test

**: The summary score for SF–36 represents the heal related to the item, so the higher score represents better conditions.

**Fig. S1**

**
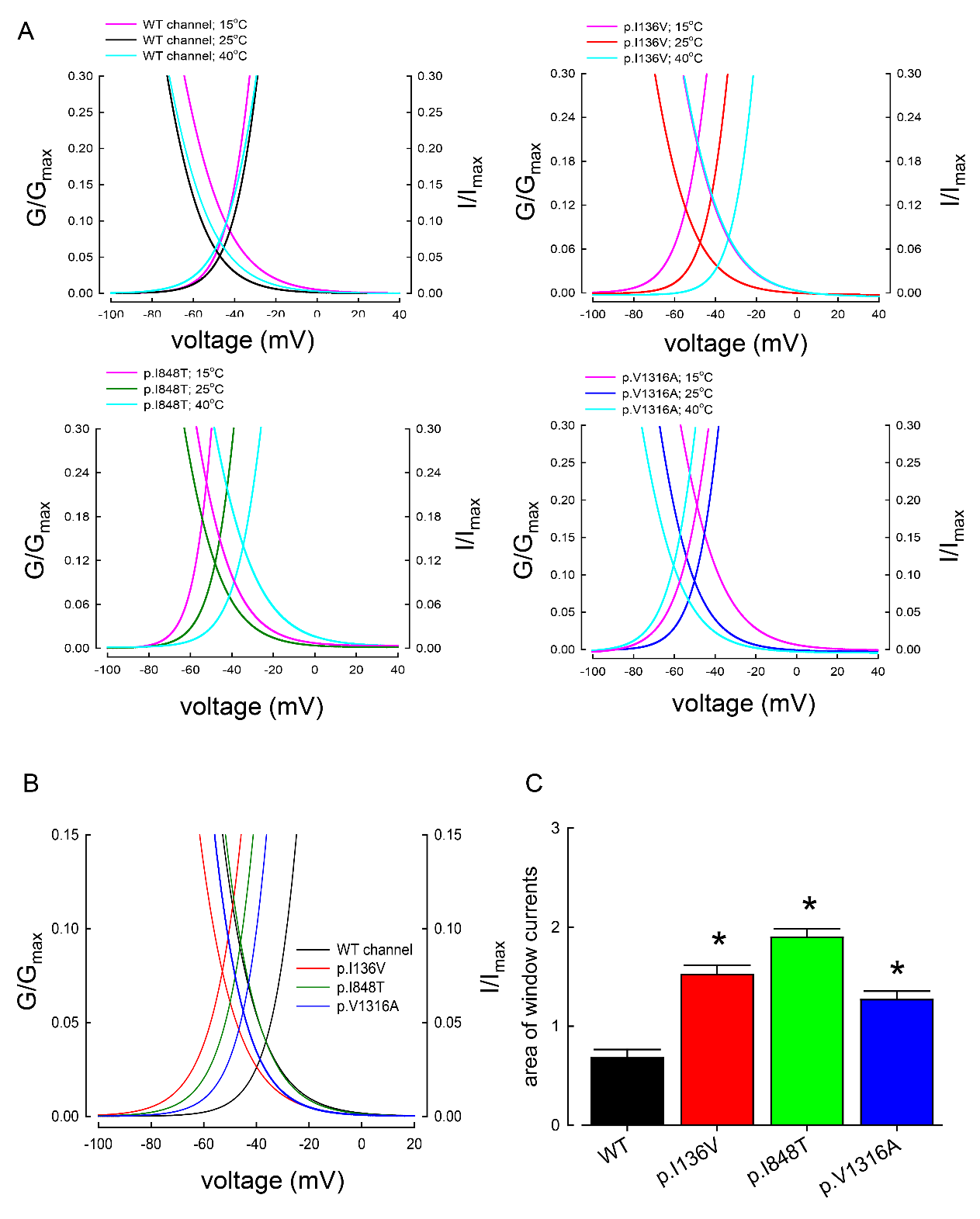
**

**Fig. S1 The activation and inactivation curves of WT, p.I136V, p.I848T, and p.V1316A mutant channels in the absence of Na_v_β4 peptide**

1. A closer view of the fitting lines in Figs. 1A and Fig. 2A between –100 and +40 mV for the WT, p.I136V, p.I848T, and p.V1316A mutant channels at 15 °C, 25 °C and 40 °C, respectively
2. Cumulative results obtained from the same protocols in Fig. 4A for the WT, p.I136V, p.I848T, and p.V1316A mutant channels in the absence of Na_v_β4 peptide. A closer view of the fitting lines between –100 and +20 mV.
3. The area of window currents obtained from the part **B** for the WT, p.I136V, p.I848T, and p.V1316A mutant channels. (*, p<0.05 between the WT and each mutant channel).

**Fig. S2**

**
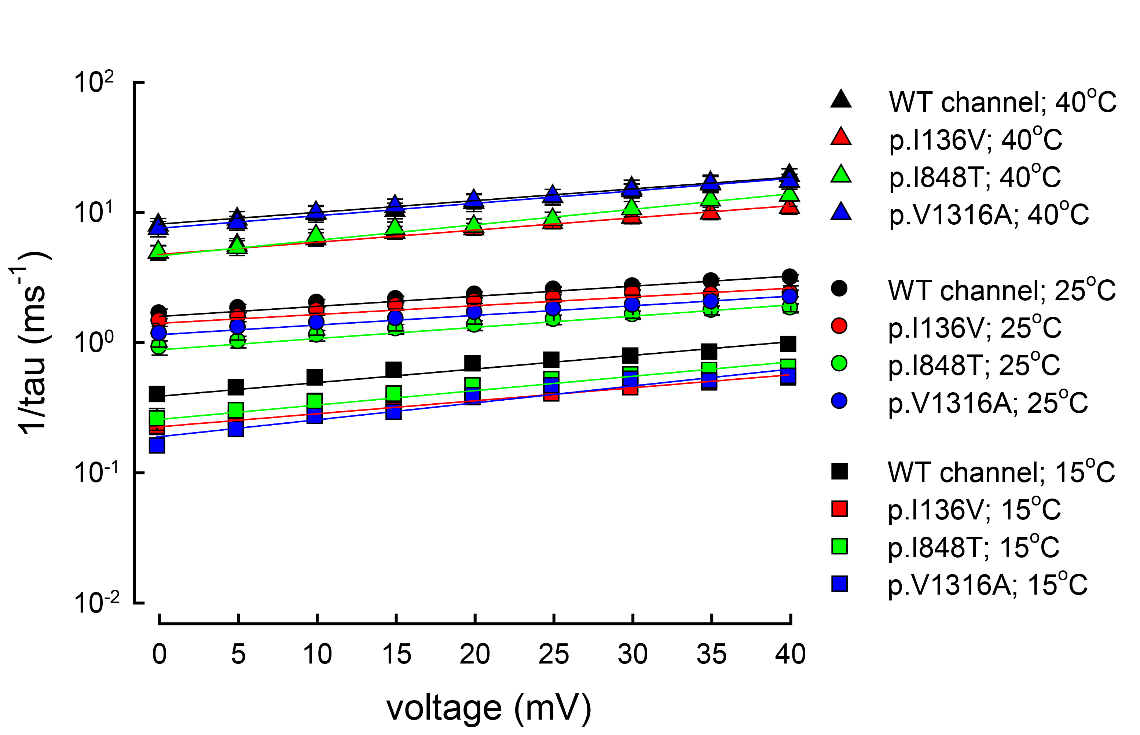
**

**Fig. S2 Inactivation kinetics in the WT and mutant channels at 15°C, 25°C, and 40°C**

The decay of the transient currents (Figs. 1A and Fig. 2A) at different depolarization potentials is fitted by mono–exponential functions in the WT, p.I136V, p.I848T, and p.V1316A mutant channels at 15 °C, 25 °C, and 40 °C. The lines are linear regression fits of the form: 1/tau(V)= A×exp(k×V/25) ms^-1^ where V is the membrane potential in mV. At 25 °C, A and k are 1.59±0.002 and 0.45±0.001, 1.41±0.003 and 0.39±0.001, 0.88±0.02 and 0.49±0.001, 1.15±0.002 and 0.43±0.003 for the WT, p.I136V, p.I848T, and p.V1316A mutant channels. At 15 °C, A and k are 0.38±0.001 and 0.60±0.001, 0.22±0.002 and 0.58±0.003, 0.25±0.002 and 0.64±0.003, 0.18±0.017 and 0.75±0.002 for the WT, p.I136V, p.I848T, and p.V1316A mutant channels. At 40 °C, A and k are 7.93±0.003 and 0.52±0.002, 4.63±0.001 and 0.54±0.001, 4.53±0.001 and 0.69±0.001, 7.38±0.003 and 0.55±0.001 for the WT, p.I136V, p.I848T, and p.V1316A mutant channels.

**Fig. S3**

**
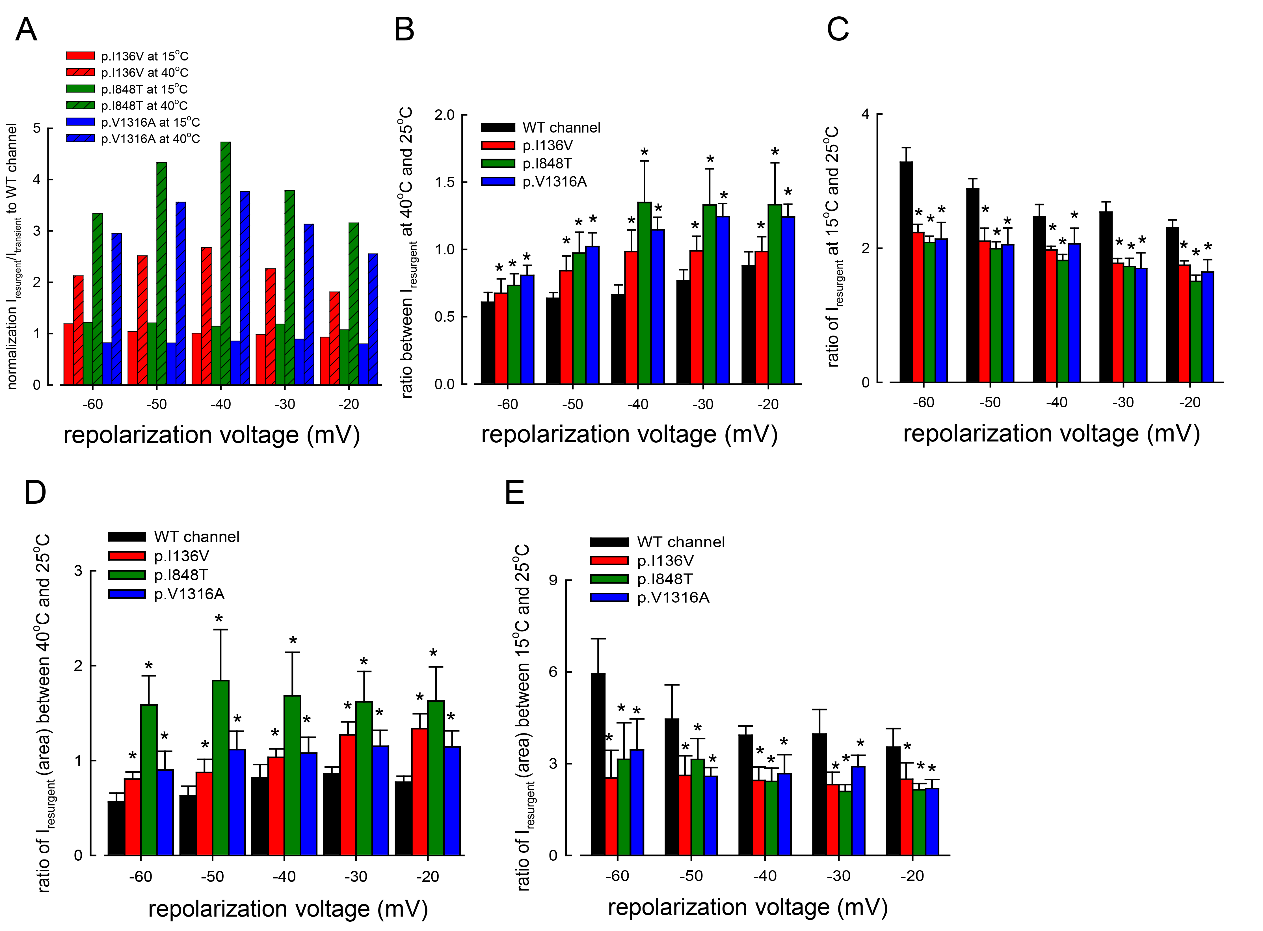
**

**Fig. S3 The amplitude of resurgent currents of WT, p.I136V, p.I848T, and p.V1316A mutant channels at 15 ^o^C, 25 ^o^C and 40 ^o^C**

1. The ratio between resurgent and transient currents is normalized to that of the WT channel for each different mutant channels. The normalized ratio is always larger at 40 ^o^C than at 15 ^o^C in the mutant channels.
2. Cumulative results were obtained from the experiments described in part A. The ratio between the resurgent currents at 40 ^o^C and 25 ^o^C in the same cell are always larger in the mutant than in the WT channels (n= 3‒6 for each measurement). *, p<0.05.
3. Cumulative results were obtained from part **A**. The ratios of resurgent currents at 15^o^C and 25^o^C in the same cell are smaller in the mutant than in the WT channel (n= 5 for each measurement). *, p<0.05.
4. Cumulative results were obtained from the experiments described in Fig. 3A. The ratio between the area under resurgent currents (the total charges transfer with resurgent currents) at 40^o^C and that at 25^o^C is plotted against the repolarization voltage between –20 and –60 mV. The ratio is significantly larger in the p.I136V, p.I848T and p.V1316A mutant than in the WT channels (n= 3–5 for each measurement). *, p<0.05
5. Cumulative results were obtained from the experiments described in Fig. 3A. The ratio between the area under resurgent currents (the total charges transfer with resurgent currents) at 15^o^C and that at 25^o^C is plotted against the repolarization voltage between –20 and –60 mV. The ratio is significantly larger in the WT channel than in the p.I136V, p.I848T and p.V1316A mutant channels (n= 5 for each measurement). *, p<0.05

**Fig. S4**

**
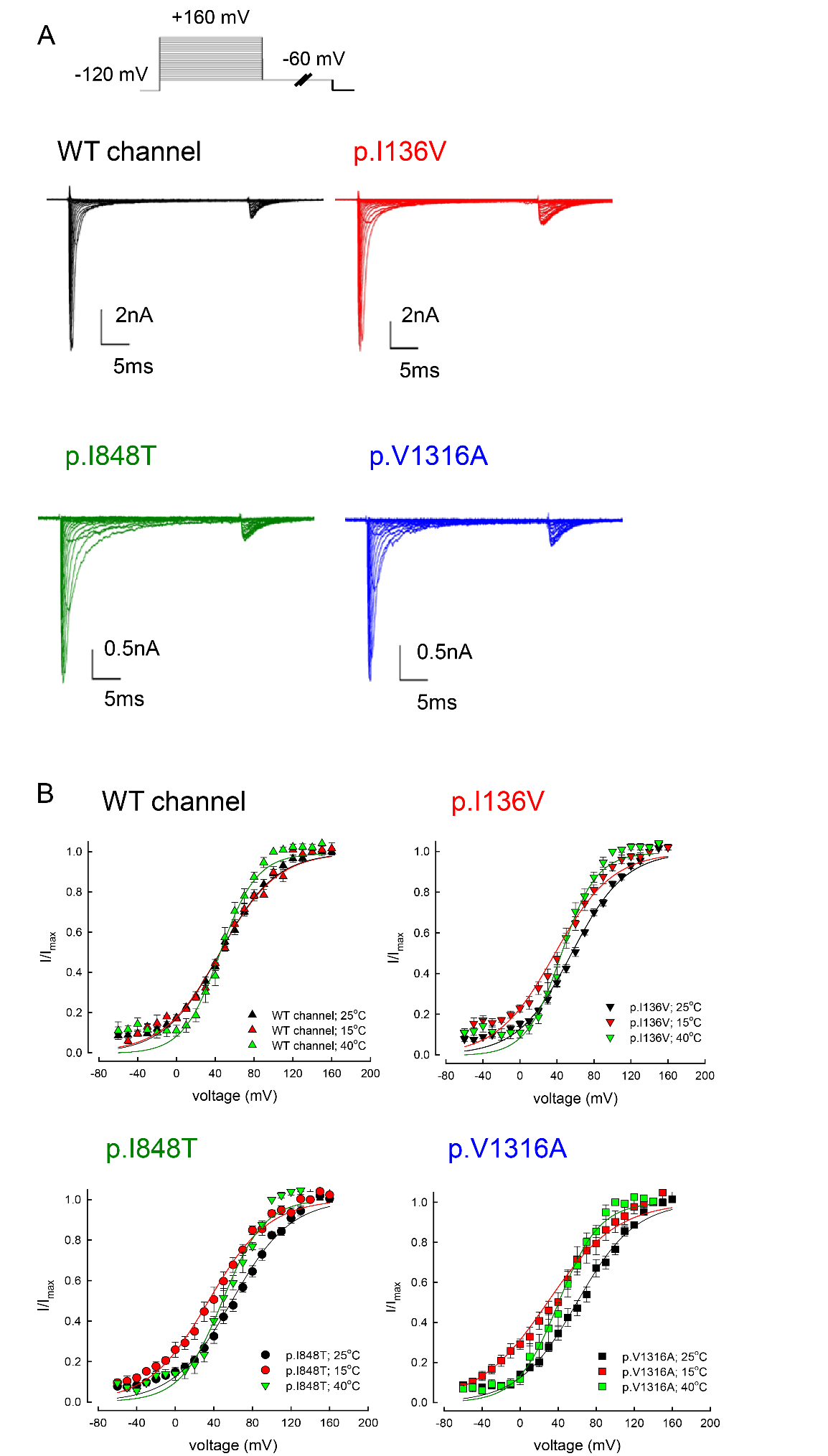
**

**Fig. S4 The activation curves of resurgent currents in the WT, p.I136V, p.I848T, and p.V1316A mutant channels at 15°C, 25 °C, and 40 °C**

1. The cell was first held at –120 mV for ~30 ms, and stepped to depolarization prepulse between –60 and +180 mV for 30 ms in 10 mV increment. Resurgent currents were then evoked by a pulse to –60 mV for 150 ms.
2. The amplitude of the resurgent currents is normalized to that evoked by a prepulse to +160 mV in the same series and plotted against the prepulse voltage to make the activation curves of resurgent currents for the WT, p.I136V, p.I848T, and p.V1316A mutant channels at 15 ^o^C, 25 ^o^C, and 40 ^o^C. The curves are fitted with a Boltzmann function of the form: 1/[1+exp((V_h_–V)/*k*)], where V is the membrane potential in mV. In the WT channel, V_h_ and *k* are 44.8±2.2 mV and 29.3±1.3, 44.5±2.5 mV and 28.6±1.5 and 44.7±1.9 mV and 19.1±1.5 at 15 ^o^C, 25 ^o^C, 40 ^o^C, respectively. In the p.I136V mutant channel, V_h_ and *k* are 37.6±2.1 mV and 17.8±1.2, 45.8±2.2 mV and 29.0±1.8 and 44.7±2.9 mV and 19.1±1.9 at 15 ^o^C, 25 ^o^C, 40 ^o^C, respectively. In the p.I848T mutant channel, V_h_ and *k* are 34.6±1.9 mV and 31.1±1.4, 58.8±2.17 mV and 30.3±1.13 and 49.1±2.1 mV and 21.8±1.2 at 15 ^o^C, 25 ^o^C, 40 ^o^C, respectively. In the p.V1316A mutant channel, V_h_ and *k* are 38.8±2.0 mV and 36.7±2.3, 45.8±2.5 mV and 30.3±1.9 and 40.8±2.3 mV and 21.7±2.0 at 15 ^o^C, 25 ^o^C, 40 ^o^C, respectively (n= 5 for each measurement).

**Fig. S5**


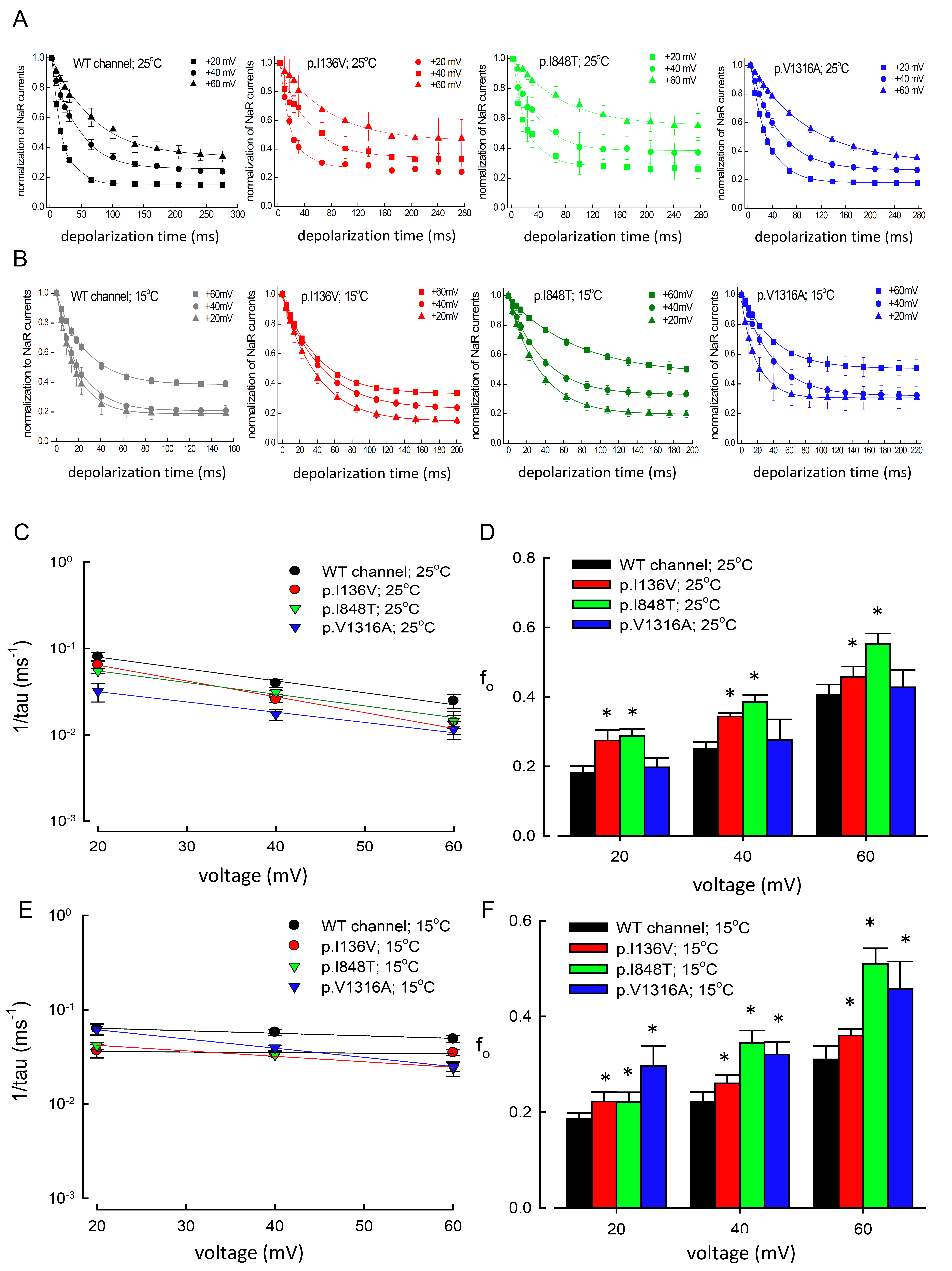


**Fig. S5 Decrease of resurgent currents with lengthening of the depolarization prepulse in the WT, p.I136V, p.I848T, and p.V1316A mutant channels at 15 ^o^C and 25 ^o^C**

1. Resurgent currents were elicited by a pulse at –60 mV following a prepulse to +20, +40, and +60 mV of different lengths. The amplitude of resurgent currents (normalized to the first one in the same series) is plotted against the length of the prepulse (e.g., +20, +40, and +60 mV) at 25 ^o^C. The lines are fits to the data points of the form: normalized resurgent currents= (1–f_0_)×exp [–(x–4.5)/τ)]+f_0_, where x is the prepulse length in ms. In the WT channel, τ and f_0_ are 13±2.2 ms and 0.17±0.02, 30.5±1.4 ms and 0.3±0.02, and 64.2±1.9 ms and 0.4±0.03 for +20, +40, and +60 mV prepulse, respectively. In the p.I136V mutant channel, τ and f_0_ are 15.5±1.4 ms and 0.27±0.03, 39.1±2.5 ms and 0.34±0.01, 70.4±1.6 ms and 0.45±0.03 for +20, +40, and +60 mV prepulse, respectively. In the p.I848T mutant channel, τ and fo are 18.3±2.0 ms and 0.28±0.02, 32.1±1.76 ms and 0.38±0.02, 69.8±0.15 ms and 0.55±0.03 for +20, +40, and +60 mV prepulse, respectively. In the p.V1316A mutant channel, τ and f_0_ are 32.0±1.5 ms and 0.2±0.27, 57.0±1.2 ms and 0.3±0.06, 86±1.7 ms and 0.4±0.05 for +20, +40, and +60 mV prepulse, respectively.
2. Similar experiments to that in part **A** were repeated at 15 ^o^C. In the WT channel, τ and fo are 17.6±2.6 ms and 0.22±0.012, 17.9±1.31 ms and 0.2±0.02, 21.4±2.0 ms and 0.4±0.02 for +20, +40, and +60 mV prepulse, respectively. In the p.I136V mutant channel, τ and f_0_ are 32.1±2.5 ms and 0.2±0.02, 32.8±2.13 ms and 0.26±0.02, 29.4±2.2 ms and 0.36±0.01 for +20, +40, and +60 mV prepulse, respectively. In the p.I848T mutant channel, τ and f_0_ are 25.1±2.2 ms and 0.22±0.02, 30.6±1.84 ms and 0.34±0.02, 53.6±2.7 ms and 0.5±0.03 for +20, +40, and +60 mV prepulse, respectively. In the p.V1316A mutant channel, τ and f_0_ are 18.9±3.6 ms and 0.3±0.04, 27.7±1.6 ms and 0.32±0.02, 41.2±2.01 ms and 0.4±0.05 for +20, +40, and +60 mV prepulse, respectively (n = 4–5 for each measurement).
3. The inverses of time constants in part **A** are plotted against the prepulse voltage in semi–logarithmic scale. The data are fitted with the following equation 1/tau_(v)_= 0.15±0.02×exp(–0.8±0.01V/25) ms^–1^ for the WT channel, 1/tau_(v)_= 0.15±0.01×exp(–0.9±0.01V/25) ms^–1^ for the p.I136V mutant channel, 0.103±0.01×exp(–0.78±0.012V/25) ms^–1^ for the p.I848T mutant channel, and 0.05±0.02×exp(–0.69±0.013V/25) ms^–1^ for the p.V1316A mutant channel, where V is the prepulse potential in mV.
4. The f_0_ (the residual resurgent Na^+^ currents) in part **A** are plotted against depolarization potentials (e.g., +20 mV, +40 mV, and +60 mV) in the WT, p.I136V, p.I848T, and p.V1316A mutant channels, respectively. *, p<0.05.
5. The inverses of time constants in part **B** are plotted against the prepulse voltage in semi–logarithmic scale. The data are fitted with the following equation 1/tau_(v)_= 0.07±0.01×exp(–0.155±0.01V/25) ms^–1^ for the WT channel, 0.036±0.01×exp(–0.03±0.012V/25) ms^–1^ for the p.I136V mutant channel, 0.097±0.02×exp(–0.56±0.02V/25) ms^–1^ for the p.I848T mutant channel, and 0.005±0.02×exp(–0.34±0.01V/25) ms^–1^ for the p.V1316A mutant channel, where V is the prepulse potential in mV. It is of note that the decrease of resurgent currents is slightly slower in the mutant than in the WT channel. The difference, however, remains similar at 15 ^o^C and 25 ^o^C.
6. The f_0_ (the residual resurgent currents) in part **B** are plotted against depolarization potentials (e.g., +20 mV, +40 mV, and +60 mV) in the WT, p.I136V, p.I848T, and p.V1316A mutant channels. *, p<0.05.

**Fig. S6**

**Fig. S6 The recovery time courses at recovery voltage of –120mV from the WT p.I136V, p.I848T, and p.V1316A mutant channels at 15 ^o^C, and 25 ^o^C**

1. The cell was held at –120 mV and pulsed twice to +20 mV (each for 10 ms) every 1.5 sec, with a gradually lengthened gap between the two pulses at –120 mV (the recovery voltage, V_r_). The sweeps are arranged so that the currents in the second pulse are gradually shifted rightward as the gap is lengthened (by 0.1 ms between each sweep).
2. Recovery time courses were obtained with experiments in part **A** (each n = 4–5). Fraction recovered is defined as the ratio between the peak current in the second pulse and that in the first pulse, and is plotted against the duration of V_r_ to make the time course of recovery form inactivation. Note the lack of discernible difference among the WT, p.I136V, p.I848T, and p.V1316A mutant channel at either 25 ^o^C or 15 ^o^C.
3. The time courses of recovery from inactivation in part B are fitted with a mono–exponential equation. Cumulative results of the time constants from the fits (each n= 4–5 for measurement) show that the recovery from inactivation at –120 mV is rather similar in all channels at the two different temperatures (*, p< 0.05).

**Fig. S7**

**
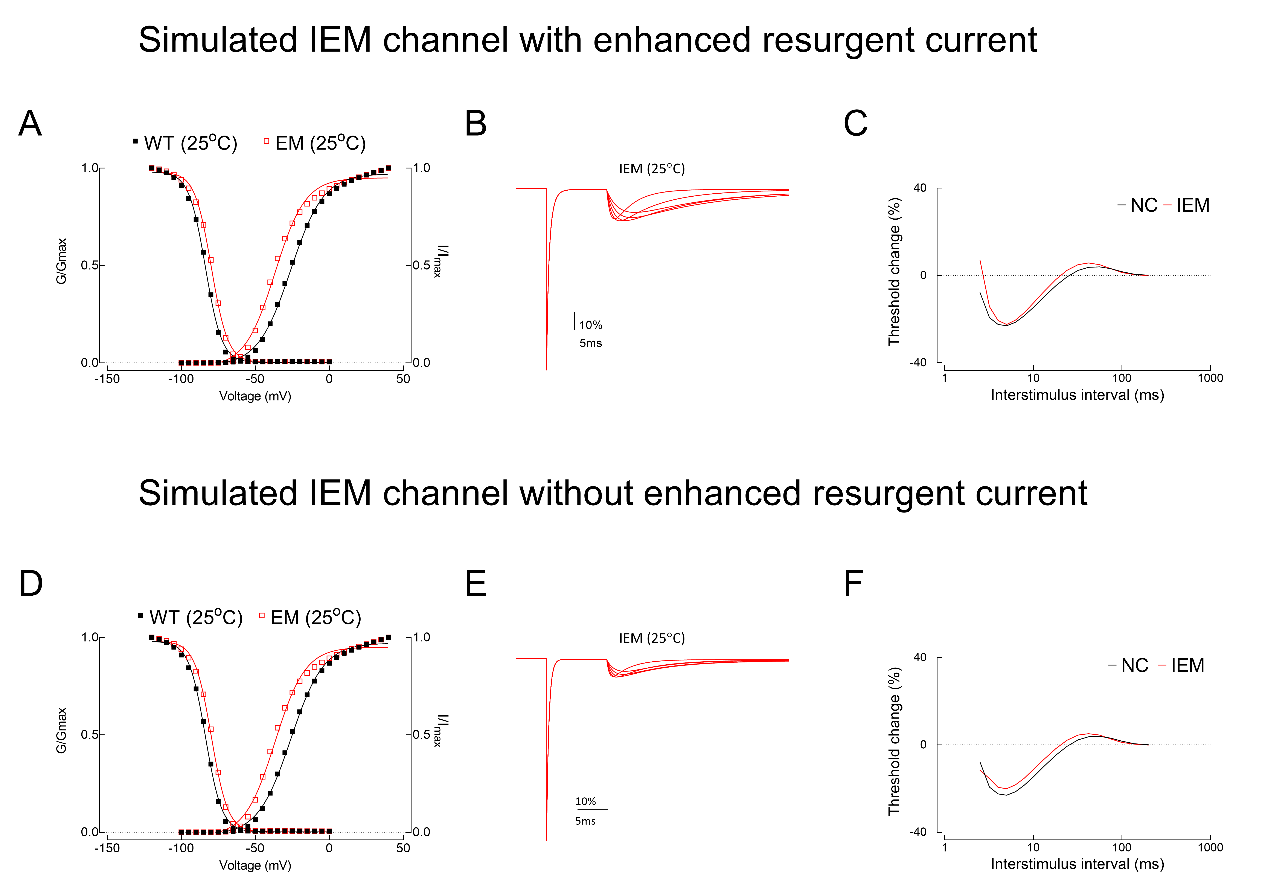
**

**Fig. S7 Simulated sodium current and recovery cycle in sensory axon, with and without enhanced resurgent current**

**(A), (B), and (C)** Activation and inactivation curve, sampled sweep of resurgent current and recovery cycle simulated with the same kinetic parameters for IEM channel shown in Fig. 6

**(D), (E), and (F)** Activation and inactivation curve, sampled sweep of resurgent current and recovery cycle simulated with a hypothetical IEM channel with only change in α (from *100*·*e^0.08*E^* to *500*·*e^0.08*E^* where *E* is membrane potential) and β (*0.55*·*e^-0.01*E^* to *4*·*e^-0.01*E^*) to demonstrate the effect of resurgent current on the excitability change.

**Mathematical model for human myelinated axon**

**Rate and voltage constants for Hodgkin**–**Huxley type channel models**

|  | A (ms^-1^, at 301.8 K) | Q_10_ | B (mV) | C (mV) |
| --- | --- | --- | --- | --- |
| α_m_ | 3.482 | 2.2 | –18.5 | 10.3 |
| β_m_ | 0.161 | 2.2 | –22.8 | 9.16 |
| α_mp_ | 1.741 | 2.2 | –36.5 | 10.3 |
| β_mp_ | 0.0805 | 2.2 | –40.8 | 9.16 |
| α_h_ | 0.05615 | 2.9 | –115.1 | 13.6 |
| β_h_ | 3.843 | 2.9 | –32.9 | 16.567 |
| α_n_ | 0.021 | 3 | –90.8 | 11 |
| β_n_ | 0.0373 | 3 | –73.6 | 10.5 |
| α_s_ | 0.002814 | 3 | –22.5 | 14.6 |
| β_s_ | 0.001704 | 3 | –90.1 | 13.49 |
| α_q_,β_q_ | 0.00192 | 3 | –85.0 | –13.7 |

$$\frac{dE}{dt}=-\frac{\left( I_{Na}+I_{Kf}+I_{Ks}+I_{Lk}+I_{pump}+I_{external}+I_{BB} \right)}{\left( C_{n}+C_{m} \right)}$$

$$\frac{dE_{i}}{dt}=-\left( I_{Kfi}+I_{Ksi}+I_{h}+I_{pumpi}+I_{Lki}-I_{BB}-C_{m}\times\frac{dE}{dt} \right)/{C_{i}}$$

$$I_{Na}=\frac{P_{Na}\frac{EF^{2}}{RT}\left( {Sel}_{Na}\left( {Na}_{o}-{Na}_{i}e^{\frac{EF}{RT}} \right)+\left( 1-{Sel}_{Na} \right)\left( K_{o}-K_{i}e^{\frac{EF}{RT}} \right) \right)}{\left( 1-e^{\frac{EF}{RT}} \right)}\times\left( \left( 1-p_{{Na}_{v}1.7} \right){\cdot m}^{3}h+p_{{Na}_{v}1.7}\cdot\left( O_{1}+O_{2} \right) \right)$$

$$I_{Nap}=\frac{P_{Na}\frac{EF^{2}}{RT}\left( {Sel}_{Na}\left( {Na}_{o}-{Na}_{i}e^{\frac{EF}{RT}} \right)+\left( 1-{Sel}_{Na} \right)\left( K_{o}-K_{i}e^{\frac{EF}{RT}} \right) \right)}{\left( 1-e^{\frac{EF}{RT}} \right)}\times P_{Nap}m_{p}^{3}$$

$$I_{Kf}=G_{Kf}n^{4}\left( E-E_{Kf} \right)$$

$$I_{Kfi}=G_{Kfi}n_{i}^{4}\left( E_{i}-E_{Kf} \right)$$

$$I_{Ks}=G_{Ks}s\left( E-E_{Ks} \right)$$

$$I_{Ksi}=G_{Ksi}s_{i}\left( E_{i}-E_{Ks} \right)$$

$$I_{h}=G_{h}q\left( E_{i}-E_{h} \right)$$

$$I_{BB}=G_{BB}\left( E-E_{i} \right)$$

$$I_{Lk}=G_{Lk}\left( E-E_{resting} \right)$$

$$I_{Lki}=G_{Lki}\left( E_{i}-E_{resting,i} \right)$$

$$E_{x}=\frac{ln\left( \frac{K_{o}+{Sel}_{x}{Na}_{o}-{Sel}_{x}K_{o}}{K_{i}+{Sel}_{x}{Na}_{i}-{Sel}_{x}K_{i}} \right)}{FRT}, for x=K_{f},K_{s},h$$

$$\frac{dx}{dt}=\alpha_{x}\left( 1-x \right)-\beta_{x}x, x for m,m_{p},h,n,s,n_{i},s_{i},q$$

$$\alpha_{m},\alpha_{m_{p}},\alpha_{n},\alpha_{s}=\frac{A\left( E-B \right)}{1-e^{\left( B-E \right)/C}}$$

$$\alpha_{h},\beta_{m},\beta_{m_{p}},\beta_{n},\beta_{s}=\frac{A\left( B-E \right)}{1-e^{\left( E-B \right)/C}}$$

$$\beta_{h}=\frac{A}{\left( 1+e^{\left( B-E \right)/C} \right)}$$

$$\alpha_{q}=Ae^{\left( E-B \right)/C}$$

$$\beta_{q}=\frac{A}{e^{\left( E-B \right)/C}}$$

$$\frac{dA_{Na}}{dt}=Q_{Na}\times A_{Na}$$

${Sel}_{Na}={0.9, Sel}_{Kf}{=0, Sel}_{Ks}{=0, Sel}_{h}=0.097$.

$$P_{Na}=4.05 {cm}^{3}s^{-1}\times{10}^{-9},P_{Nap}=1\%,G_{Ks}=41 nS,G_{Ksi}=0.365 nS,$$

$$G_{Kf}=20 nS,G_{Kfi}=100 nS,G_{Lk}=1.68 nS,G_{Lki}=1.6 nS,G_{h}=6.3 nS,$$

$G_{BB}=35 nS,I_{pump}=30 pA,I_{pumpi}=30 pA$.

$C_{n}{=1.5pF, C}_{m}{=1.55pF, C}_{i}=290pF$.

$${F=96485 C}/{mol}, R=8314 {{cm}^{3}kPa}/{Kmol,} T=308K.$$

C_n_: nodal capacitance, C_m_: myelin capacitance, C_i_: internodal capacitance, E: nodal potential, E_i_: internodal potential, E_resting_: resting nodal potential, E_resting,i_=resting internodal potential, E_x_: reversal potential for current x, P_Na_ and I_Na_: transient sodium permeability and current, I_Nap_ = persistent sodium current, G_Ks_ and I_Ks_: nodal slow potassium conductance and current, G_Kf_ and I_Kf_: nodal fast potassium conductance current, G_Ksi_ and I_Ksi_: intermodal slow potassium current, G_Kfi_ and I_Kfi_: intermodal fast potassium conductance current, G_h_ and I_h_: hyperpolarization–activated cyclic nucleotide–gated (HCN) conductance and current, G_BB_ and I_BB_: Barrett–Barrett conductance and current, G_LK_ and I_LK_: nodal leak conductance and current, G_LKi_ and I_LKi_: internodal leak conductance and current, F: Faraday constant, T: temperature, in K, R: Gas constant, Sel_x_: selectivity of ion x, A_Na_: probability vector of Na_v_1.7 current, Q_Na_: rate matrix of Na_v_1.7 current, p_Nav1.7_:percentage of Na_v_1.7 in total sodium conductance.
